# Integrated analysis of fecal microbiome and serum metabolome reveals the profiling of gut microbiota-related metabolites in rats and mice subjected to prolonged exposure to a high-humidity environment

**DOI:** 10.3389/fcimb.2026.1782615

**Published:** 2026-06-22

**Authors:** Hexin Zhu, Shumin Wei, Haixia Liu, Hua Yang, Tong Liu, Wei Jiang, Weihui Lu, Taohua Lan

**Affiliations:** 1 The Second Clinical Medical College of Guangzhou University of Chinese Medicine, Guangzhou, China; 2 State Key Laboratory of Dampness Syndrome of Chinese Medicine, The Second Affiliated Hospital of Guangzhou University of Chinese Medicine, Guangzhou, China; 3 Department of Cardiology, Guangdong Provincial Hospital of Chinese Medicine, Guangzhou, China; 4 State Key Laboratory of Traditional Chinese Medicine Syndrome, Guangdong Provincial Hospital of Chinese Medicine, Guangzhou University of Chinese Medicine, Guangzhou, China; 5 Chinese Medicine Guangdong Laboratory, Guangdong, Hengqin, China

**Keywords:** 16S rRNA sequencing, environment, gut microbiota, high humidity, metabolites

## Abstract

**Background:**

High humidity, as a key climate risk factor, has become one of the significant threats to public health. However, less is known about the mechanism by which the high-humidity environment affects the health of the population. The present study was designed to reveal the profile of gut microbiota-related metabolites in rats and mice subjected to prolonged exposure to a high-humidity environment.

**Methods:**

Sprague–Dawley rats and C57BL/6 mice were housed under standard conditions (relative humidity of 60% ± 5%) or prolonged exposure to a high-humidity environment (relative humidity of 90% ± 5%) for 7, 14, and 28 days, respectively. Integrated analysis of fecal microbial diversity and serum metabolome was performed using 16S rRNA sequencing and non-targeted metabolomics with LC-MS/MS.

**Results:**

High-humidity exposure led to significant changes in the composition of the gut microbiota and serum metabolic profiles in both rat and mouse models. Our results revealed that disorders in glycerophospholipid metabolism, ABC transporters, and phenylalanine metabolism are key metabolic characteristics of hyperhumidity exposure. In addition, multi-omics correlation analysis identified the key gut microbiota-related metabolites, including phosphocholine, choline, LPC(16:0), taurine, L-valine, L-proline, 2-hydroxycinnamic acid, phenylacetaldehyde, P-salicylic acid, and PC(16:0/20:4(5Z,8Z,11Z,14Z)), which contributed to the pathogenic effect of high humidity.

**Conclusions:**

The present study revealed that high-humidity exposure disrupts the host’s metabolic homeostasis by altering the gut microbiota-related metabolites in rat and mouse models, showing commonalities and specificities. Our findings may provide new ideas and insights for further study on the pathogenic mechanism of hyperhumidity and intervention strategies targeting the microbiota.

## 1 Introduction

It is well known that environmental risk factors, such as climate change, are becoming a major public health concern (Vecellio et al., 2023). Multiple studies showed that extreme humidity is associated with higher risks of mortality throughout the world, especially with the exacerbating risk of cardiovascular diseases (Dimitrova et al., 2021). Studies have shown that compared to normal humidity days, the emergency risk of high-temperature-related cardiovascular diseases increases by six times when the relative humidity exceeds 82% (Rahman et al., 2025). In summer, every 10% increase in humidity is accompanied by a rise in the risk of cardiovascular diseases by 17% (Zheng et al., 2024). Extreme high humidity is also closely related to an increased risk of death from respiratory diseases (Chen et al., 2021).

The integrated analysis of the microbiome and metabolome is a key strategy for systematically understanding the host–microbiome co-metabolic network and precisely evaluating the effects of intervention (Wang et al., 2025). By correlating the composition of microorganisms with changes in their metabolic products, it is possible to reveal the functional activity of the microbiota and its regulatory mechanisms on host physiology (Freire-Zapata et al., 2024), providing a multidimensional perspective for understanding health imbalances caused by environmental stress. Studies have shown that environmental stress, especially heat stress, significantly alters the diversity of the microbiota and the abundance of dominant bacterial genera by changing the pH value of the intestinal microenvironment and disrupting the intestinal epithelial barrier (Wang et al., 2026). Heat stress may lead to a reduction in beneficial bacteria and promote an increase in pro-inflammatory bacteria in the intestines of fish, thereby triggering metabolic disorders and intestinal inflammation (Zhou et al., 2022). Dysbiosis can affect the functions of multiple organs through abnormal release of metabolic products and induction of low-grade inflammation (Kvitne et al., 2026). Bile acids derived from the microbiota can regulate the FXR signaling pathway and participate in the regulation of metabolic homeostasis (Won et al., 2025). Recent research has found that high humidity directly disrupts the structure of the intestinal microbiota (Wu et al., 2024), indicating that the imbalance of the microbiota may be involved in the pathogenic mechanism of high humidity. However, the impact of high-humidity exposure on the microbiota–metabolic axis to host health, especially in different times of exposure and in different animals, has not been fully clarified.

In this study, an integrative analysis of 16S rRNA sequencing and LC-MS non-targeted metabolomics was adopted to reveal the profile of gut microbiota-related metabolites in rats and mice subjected to prolonged exposure to a high-humidity environment.

## 2 Materials and methods

### 2.1 Animal model and grouping

Male Sprague–Dawley rats (specific pathogen-free, weighing 250 to 280 g) and C57BL/6 mice (specific pathogen-free, weighing 20 to 23 g) were obtained from the Beijing HFK Bioscience Co., Ltd. Animals were housed under standard conditions with 12-h light/dark cycles, constant room temperature of 22 °C ± 2 °C, and relative humidity of 60%–65%. After 1 week of adaptive feeding with a standard lab diet, animals were randomized into a control group and a dampness group (*n* = 6). Rats and mice in the dampness group were exposed to high-humidity conditions (temperature 22 °C ± 2 °C, relative humidity 90%–95%) via an artificial climate box (model: LRH-600A-HS, Guangdong Taihongjun Scientific Instrument Co., Ltd., China) for 7, 14, and 28 days, respectively. Control rats and mice were housed under standard conditions without any treatment. All animals were provided with fresh feed supplies every day.

### 2.2 16S rRNA sequencing

Fecal samples were collected and snap frozen in liquid nitrogen within minutes of donation and then stored at −80°C for assay. Microbial diversity was detected in the fecal samples by 16S rRNA sequencing as previously described. DNA was extracted from fecal samples using the QIAamp DNA Stool Mini Kit (Qiagen, Hamburg, Germany), following the manufacturer’s instructions. The V3–V4 hypervariable regions of the bacterial 16S rRNA gene were amplified with the following primers: 338F (5′-ACTCCTACGGGAGGCAGCA-3′) and 806R (5′-GGACTACHVGGGTWTCTAAT-3′). The procedure of PCR reactions was as follows: 3 min at 95°C, 30 cycles of 30 s at 95 °C, annealing at 60 °C for 30 s, elongation at 72 °C for 75 s, and extension at 72 °C for 10 min. A triplicate mixture containing 2 μL of 10 × FastPfu buffer, 2 μL of 2.5 mM dNTPs, 0.8 μL of each primer (5 μM), 0.2 μL of FastPfu polymerase, and 10 ng of template DNA was used for the PCR reaction. Finally, we extracted the PCR products from a 2% agarose gel, which were further purified by the AxyPrep DNA Gel Extraction Kit (Axygen Biosciences, USA). DNA concentration was quantified using QuantiFluor™-ST (Promega, USA). The purified amplicons were pooled at equimolar concentrations, and further paired-end sequencing was performed using an Illumina MiSeq instrument (Illumina, San Diego, California, USA) according to standard protocols provided by Majorbio Bio-Pharm Technology Co., Ltd. Raw fastq files were quality-filtered using Trimmomatic and merged with FLASH. Operational taxonomic unit (OTU) was clustered with a 97% similarity cutoff using UPARSE (version 7.1). The taxonomy of each 16S rRNA gene sequence was assigned by the Ribosomal Database Project (RDP) Classifier algorithm against the SILVA (SSU123) 16S rRNA database using a confidence threshold of 70%.

### 2.3 Non-targeted LC-MS-based metabolic profiling

Blood samples were collected in 1.5 EP tubes and centrifuged at 1,000×*g* for 15 min at 4°C after 1 h at room temperature. Serum was separated and stored at −80°C for assay. Metabolites were detected in the serum samples by LC/MS as previously described. A UHPLC-Q Exactive mass spectrometer (Thermo Fisher Scientific, Waltham, MA, USA) was used to analyze the metabolic profiling. An ACQUITY UPLC HSS T3 column (100 mm × 2.1 mm, 1.8 um; Waters, Milford, MA, USA) was used for metabolite separation. Water (95%) and acetonitrile (5%) containing 0.1% formic acid were used as mobile phase A. Acetonitrile (47.5%), isopropanol (47.5%), and water (5%) containing 0.1% formic acid were used as mobile phase B. The linear gradient was as follows: 0.0–0.1 min at 5% B; 0.1–2 min from 5% B to 25% B; 2–9 min from 25% B to 100% B and kept for 4 min; 13–13.1 min back to 0% B and 13.1–16 min at 0% B. The flow rate was 0.4 mL/min and the column temperature was 40°C. All the samples were kept at 4°C during the analysis. The injection volume was 2 μL. Data acquisition was performed in full scan mode ranging from 70 to 1,050 (*m*/*z*), with an applied resolution of 70,000 for MS1 and 17,500 for MS2. Spray voltages (V) were set at 3,500 for positive ionization mode and 2,800 for negative ionization mode. Sheath gas and Aux gas flow rates were set at 40 and 10 arbitrary units, respectively. Capillary and auxiliary gas heater temperatures were set at 320 °C and 400 °C.

### 2.4 Statistical analysis

The data were analyzed using the R software package (V.3.2.1) and the online platform of Majorbio Cloud Platform (www.majorbio.com).

For microbiome analysis, the Shannon and Chao indices were analyzed using the Wilcoxon rank sum test to evaluate gut microbiota alpha diversity, and *p <*0.05 was considered statistically significant. Beta diversity at the OTU levels was performed by partial least squares-discriminant analysis (PLS-DA) using the Bray–Curtis distance measure. Compositional differences among groups at the genus level were tested with a non-parametric Wilcoxon rank-sum test. Linear discriminant analysis (LDA) effect size (LDA = 2, *p* < 0.05) calculated by the LEfSe software was used to determine the variation at the taxonomic level.

The pretreatment of LC/MS raw data was performed using Progenesis QI (Waters Corporation, Milford, USA) software, and a three-dimensional data matrix in CSV format was exported. The information in this three-dimensional matrix included sample information, metabolite name, and mass spectral response intensity. Internal standard peaks, as well as any known false-positive peaks (including noise, column bleed, and derivatized reagent peaks), were removed from the data matrix, deredundant, and peak-pooled. At the same time, the metabolites were identified by searching databases, and the main databases were the HMDB (http://www.hmdb.ca/), Metlin (https://metlin.scripps.edu/), and the self-compiled Majorbio Database (MJDB) of Majorbio Biotechnology Co., Ltd. (Shanghai, China). The data matrix obtained by searching the databases was uploaded to the Majorbio Cloud Platform (https://cloud.majorbio.com) for data analysis. Firstly, the data matrix was pre-processed as follows: at least 80% of the metabolic features detected in any set of the samples were retained. After filtering, the minimum value in the data matrix was selected to fill the missing value, and each metabolic signature was normalized to the sum. To reduce errors caused by sample preparation and instrument instability, the response intensities of the sample mass spectrometry peaks were normalized using the sum normalization method, to obtain the normalized data matrix. Meanwhile, the variables of the QC samples with relative standard deviation (RSD) >30% were excluded and log 10-logarithmicized, to obtain the final data matrix for subsequent analysis. Variance analysis was performed on the matrix file after data preprocessing. For metabolomics analyses, PLS-DA was carried out to visualize the metabolic alterations among the experimental groups (ropls package, R software, ver3.6.2). A heatmap was shown with red and blue indicating high and low concentrations, respectively (pheatmap package, R software, ver3.6.2). The differential metabolites were selected based on the combination of a statistically significant threshold of variable influence on projection (VIP) values obtained from the OPLS-DA model and *p*-values from a two-tailed Student’s *t*-test on the normalized peak areas, where metabolites with VIP >1.0 and *p <*0.05 were considered as differential metabolites. Differential metabolites among the two groups were mapped into their biochemical pathways through metabolic enrichment and pathway analysis based on the KEGG database (http://www.genome.jp/kegg/). These metabolites could be classified according to the pathways in which they are involved or the functions they perform. Enrichment analysis was used to determine whether a group of metabolites was represented within a functional node. This approach expands annotation analysis from individual metabolites to groups of metabolites, providing insights into their collective biological functions and pathway associations. The Python package “scipy.stats” (https://docs.scipy.org/doc/scipy/) was used to perform enrichment analysis to obtain the most relevant biological pathways for experimental treatments.

Procrustes analysis was performed to analyze the similarity and variation of samples between the microbiome and metabolome. Spearman’s correlation was used to construct a correlation heatmap between differential metabolites and various microbes.

## 3 Results

### 3.1 Gut microbiota analysis in rats with environmental high humidity

**Figure 1:**
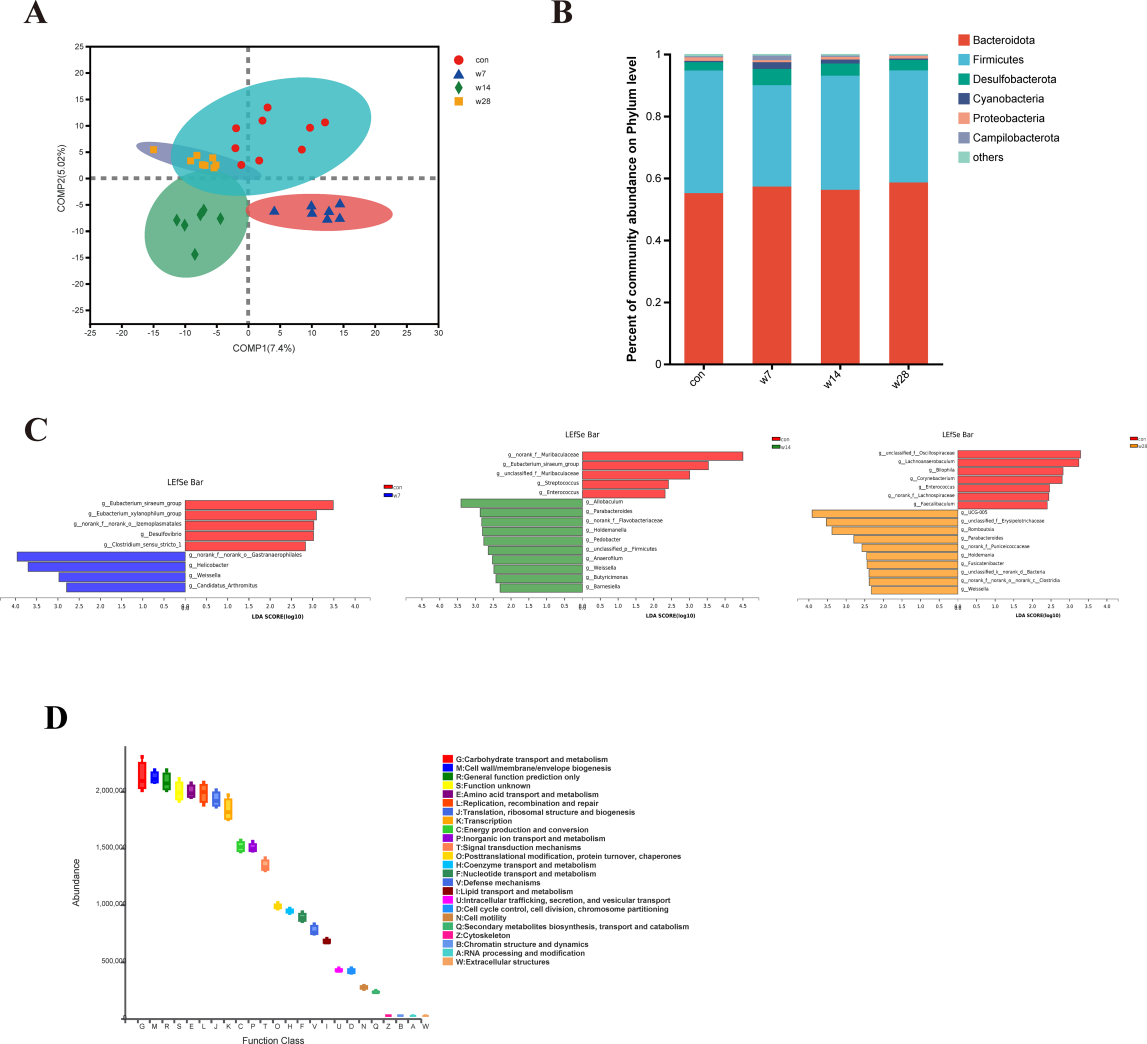
Gut microbiota analysis in rats with environmental high humidity. **(A)** Score plots of partial least squares-discriminant analysis (PLS-DA) at the OTU level. One dot in the figure represents one sample. **(B)** The composition and relative abundance of fecal microbiota at the phylum level. **(C)** Linear discriminant analysis (LDA) effect size (LEfSe) bar plot. **(D)** The main COG functions of gut microbiota in the control and dampness groups. con, control group; w7, w14, and w28 indicate groups housed under high-humidity conditions for 7, 14, and 28 days, respectively.

Microbial diversity was detected in the fecal samples from the control and dampness rats (rats in the W7, W14, and W28 groups) by 16S rRNA sequencing. Alpha diversity analysis revealed no significant difference in gut microbiota diversity between the control and dampness rats based on the Shannon and Chao indices (*p* > 0.05) (as shown in **Supplementary Figure 1**). The results of beta diversity at the OTU level based on PLS-DA showed apparent separations in the microbial composition between the control and dampness groups (**Figure 1A**). The relative abundance percentage of gut microbiota at the phylum level showed that the dominant phyla in each group were Bacteroidetes and Firmicutes (**Figure 1B**). Rats in the W7, W14, and W28 groups exhibited a decreased Firmicutes/Bacteroidetes ratio compared to the control rats. LEfSe analysis was used to identify species that differed significantly between the groups. As shown in **Figure 1C**, based on LEfSe analysis with LDA >3 and *p* < 0.05, the genera enriched in the W7 group were g:Helicobacter and g:norank_f:norank_o:Gastranaerophilales, and the genus enriched in the W14 group was g:Allobaculum, while the genera enriched in the W28 group were g:UCG-005, g:Romboutsia, and g:unclassified_f:Erysipelotrichaceae. Additionally, the genera enriched in the control group were g:Eubacterium_siraeum_group, g:norank_f:norank_o:Izemoplasmatales, g:Eubacterium_xylanophilum_group, g:norank_f:Muribaculaceae, g:unclassified_f:Muribaculaceae, g:Lachnoanaerobaculum, and g:unclassified_f:Oscillospiraceae. COG enrichment analysis of gut microbiota showed that carbohydrate transport and metabolism, cell wall/membrane/envelope biogenesis, amino acid transport and metabolism, replication/recombination and repair, translation/ribosomal structure and biogenesis, transcription, inorganic ion transport and metabolism, coenzyme transport and metabolism, nucleotide transport and metabolism, and lipid transport and metabolism were mainly involved in the control and dampness groups (**Figure 1D**).

### 3.2 Serum metabolism profile in rats with environmental high humidity

**Figure 2:**
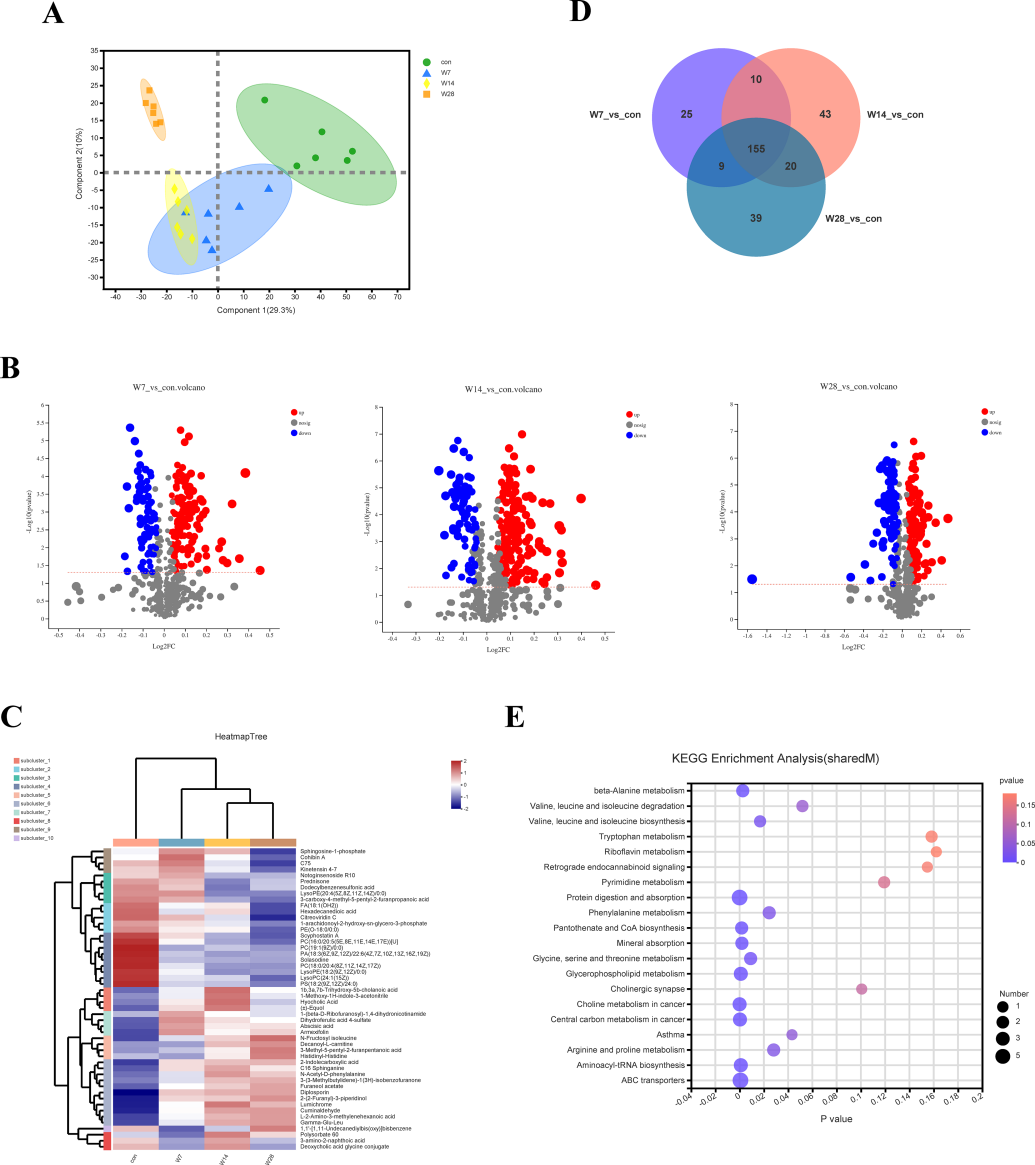
Serum metabolism profile in rats with environmental high humidity. **(A)** Score plots of partial least squares-discriminant analysis (PLS-DA). **(B)** Volcano plot of differential metabolites in the W7 vs. Con groups, W14 vs. Con groups, and W28 vs. Con groups. **(C)** Heatmap of the top 50 differential metabolites in four groups. **(D)** Venn analysis. **(E)** The bubble chart of the KEGG pathway of 155 shared differential metabolites in all three comparison groups. con, control group; w7, w14, and w28 indicate groups housed under high-humidity conditions for 7, 14, and 28 days, respectively.

Metabolites were detected in the serum samples from the control and dampness rats by LC/MS. A PLS-DA score plot was generated, and the permutation test showed that the model was reliable without overfitting [*R*^2^ = (0.0, 0.7505), Q2 = (0.0, −0.6301)] in the discovery set. As shown in **Figure 2A**, the PLS-DA plot proved the apparent separation between the control and dampness groups, implying obvious changes of the serum metabolism profile in control rats with environmental high humidity. The volcano plots showed that 127, 155, and 139 metabolites from the W7, W14, and W28 groups of dampness rats, respectively, were significantly upregulated, while 72, 73, and 84 metabolites were significantly downregulated compared with the control rats (*p* < 0.05) (**Figure 2B**). The relative concentrations of the top 50 differential metabolites in the four groups were shown in the heatmaps (**Figure 2C**), which have clear clustering and separation. As shown in **Figure 2D** and **Supplementary Table 1**, 155 differential metabolites were shared in the W7 vs. Con groups, W14 vs. Con groups, and W28 vs. Con groups. Enrichment pathway analysis of 155 shared differential metabolites in all three comparison groups is shown in **Figure 2E**, implying that dampness-induced metabolic disturbances were mainly associated with lipid metabolism (e.g., glycerophospholipid metabolism), amino acid metabolism (e.g., glycine, serine, and threonine metabolism, phenylalanine metabolism), cancers: overview (e.g., choline metabolism in cancer, central carbon metabolism in cancer), digestive system (e.g., protein digestion and absorption, mineral absorption), translation (e.g., aminoacyl-tRNA biosynthesis), membrane transport (e.g., ABC transporters), metabolism of cofactors and vitamins (e.g., pantothenate and CoA biosynthesis), and metabolism of other amino acids (e.g., beta-alanine metabolism).

### 3.3 Correlation between gut microbiota and serum metabolites in rats with environmental high humidity

**Figure 3:**
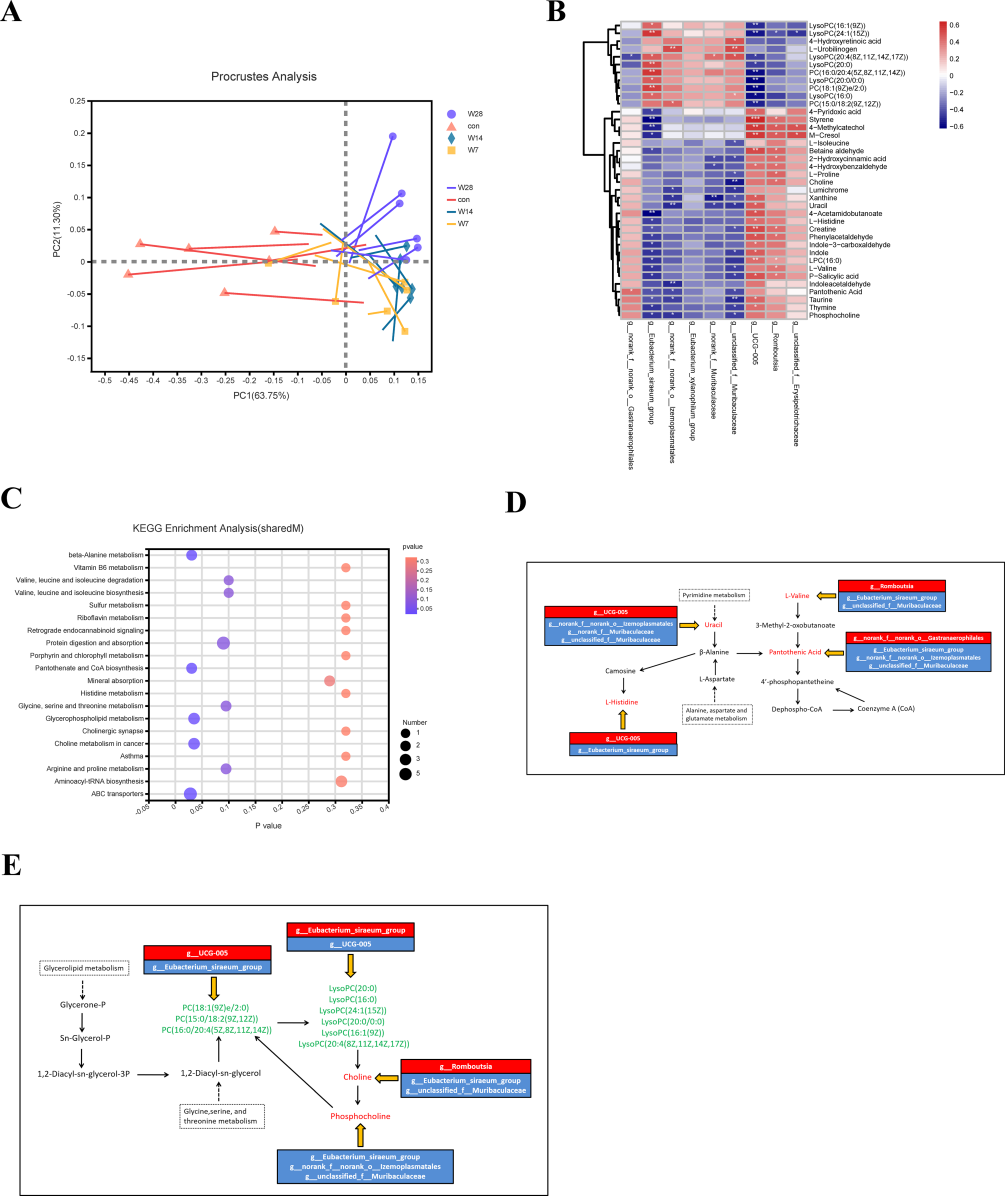
Correlation between gut microbiota and serum metabolites in rats with environmental high humidity. **(A)** Score plots of Procrustes analysis. **(B)** Correlation heatmap analysis between 9 intestinal flora and 38 microbiota-related metabolites screened based on *p <*0.05. **(C)** The bubble chart of the KEGG pathway in 38 microbiota-related metabolites. **(D)** Metabolic pathway map of pantothenate and CoA biosynthesis and beta-alanine metabolism. **(E)** Metabolic pathway map of glycerophospholipid metabolism. The metabolites expressed in red indicate statistical upregulation and those expressed in green indicate statistical downregulation in the dampness group. Other related metabolic pathways were expressed in a dotted wire frame. The intestinal flora in red boxes were positively correlated, while those in blue boxes were negatively correlated with the indicated metabolites. *p < 0.05, **p < 0.01, ***p < 0.001. con, control group; w7, w14, and w28 indicate groups housed under high-humidity conditions for 7, 14, and 28 days, respectively.

Interomics correlation analyses were used to further explore the correlation between the gut microbiota and serum metabolome. Procrustes analysis showed that the trend of microbiome abundance and metabolomics expression was significantly consistent between the control and dampness groups (*p* = 0.047) (**Figure 3A**). A correlation between 9 intestinal flora and 155 shared different serum metabolites was calculated, among which 38 microbiota-related metabolites were screened based on *p <*0.05 and illustrated in a heatmap (**Figure 3B**). The results of the metabolic pathway enrichment analysis indicated that there were five enriched pathways with significant differences between the control and dampness groups, including beta-alanine metabolism, pantothenate and CoA biosynthesis, glycerophospholipid metabolism, choline metabolism in cancer, and ABC transporters (**Figure 3C**). The pathway networks between the intestinal flora and microbiota-related metabolites enriched in pantothenate and CoA biosynthesis, beta-alanine metabolism, and glycerophospholipid metabolism were constructed. The levels of microbiota-related metabolites enriched in these three pathways are shown in **Supplementary Figure 2**.

As shown in **Figure 3D**, four microbiota-related metabolites enriched in the pantothenate and CoA biosynthesis and beta-alanine metabolic pathway (pantothenic acid, L-valine, uracil, and L-histidine) had higher concentrations in the dampness group compared with the control group. Among the nine intestinal flora, g:UCG-005 was positively correlated with uracil and L-histidine, while g:Eubacterium_siraeum_group was negatively correlated with pantothenic acid, L-valine, and L-histidine. g:norank_f:norank_o:Izemoplasmatales and g:unclassified_f:Muribaculaceae were negatively correlated with pantothenic acid and uracil.

As microbiota-related metabolites were enriched in the glycerophospholipid metabolic pathway (**Figure 3E**), choline and phosphocholine were highly expressed in the dampness group, while PCs and LysoPCs had low expression in the dampness group when compared with the control group. Among the nine intestinal flora, g:UCG-005 was positively correlated with PCs, while it was negatively correlated with LysoPCs. g:Eubacterium_siraeum_group was positively correlated with LysoPCs and was negatively correlated with PCs, choline, and phosphocholine. In addition, g:unclassified_f:Muribaculaceae was negatively correlated with phosphocholine, while g:Romboutsia was positively correlated with choline.

### 3.4 Gut microbiota analysis in mice with environmental high humidity

**Figure 4:**
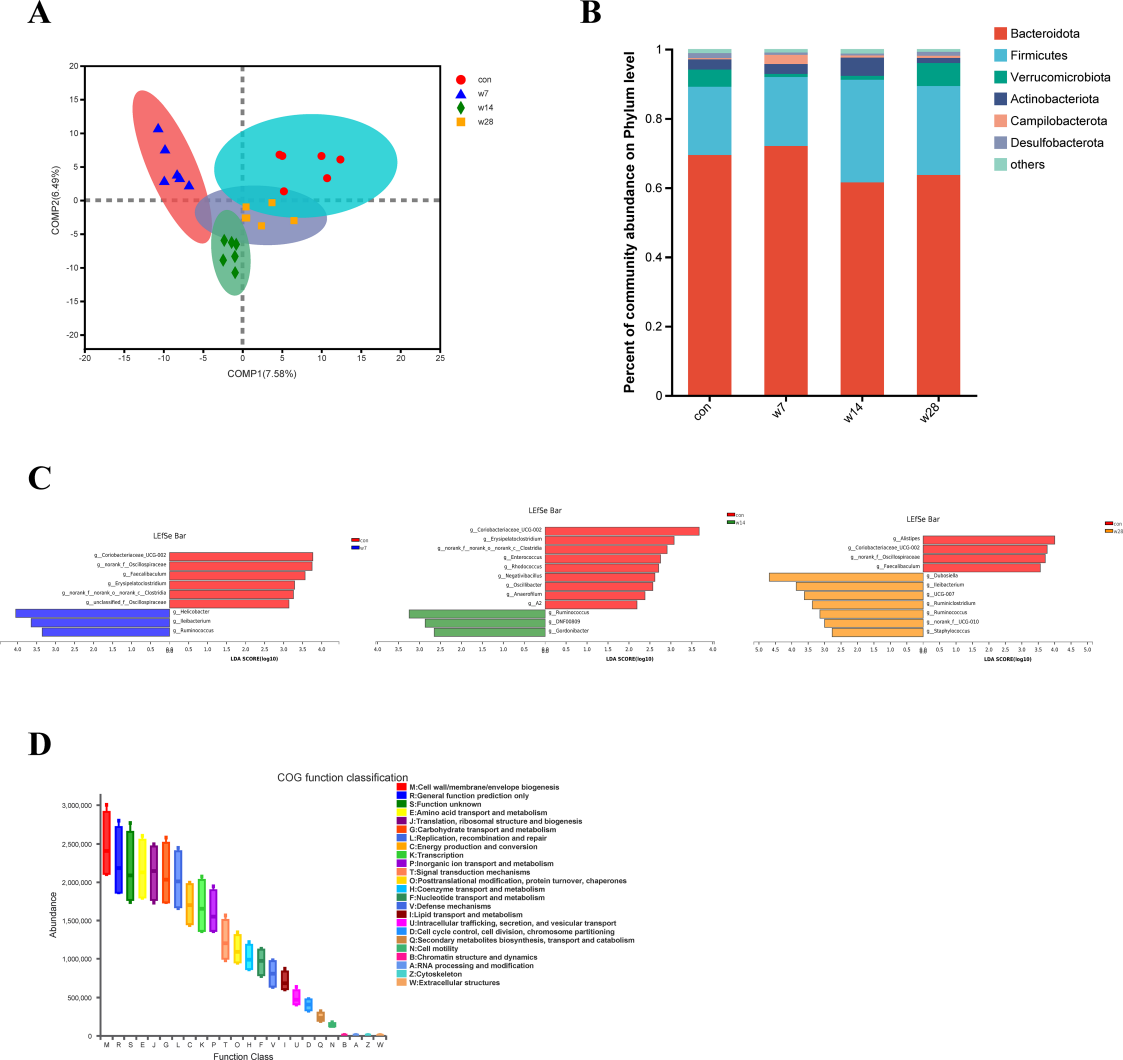
Gut microbiota analysis in mice with environmental high humidity. **(A)** Score plots of partial least squares-discriminant analysis (PLS-DA) at the OTU level. One dot in the figure represents one sample. **(B)** The composition and relative abundance of fecal microbiota at the phylum level. **(C)** Linear discriminant analysis (LDA) effect size (LEfSe) bar plot. **(D)** The main COG functions of gut microbiota in the control and dampness groups. con, control group; w7, w14, and w28 indicate groups housed under high-humidity conditions for 7, 14, and 28 days, respectively.

Microbial diversity was detected in the fecal samples from control and dampness mice (mice in the W7, W14, and W28 groups) by 16S rRNA sequencing. Alpha diversity analysis revealed no significant difference in gut microbiota diversity between control and dampness mice based on the Shannon and Chao indices (*p* > 0.05) (as shown in **Supplementary Figure 3**). The results of beta diversity at the OTU level based on PLS-DA showed apparent separation in the microbial composition between the control and dampness groups (**Figure 4A**). The relative abundance percentage of gut microbiota at the phylum level showed that the dominant phyla in each group were Bacteroidetes and Firmicutes (**Figure 4B**). Mice in the W14 and W28 groups have an increased Firmicutes/Bacteroidetes ratio compared to the control mice. As shown in **Figure 4C**, based on LEfSe analysis with LDA >3 and *p* < 0.05, the genera enriched in the W7 group were g:Ileibacterium, g:Helicobacter, and g:Ruminococcus, and the genus enriched in the W14 group was g:Ruminococcus, while the genera enriched in the W28 group were g:Ileibacterium, g:norank_f:UCG-010, g:Ruminiclostridium, g:UCG-007, g:Dubosiella, and g:Ruminococcus. Additionally, the genera enriched in the control group were g:Erysipelatoclostridium, g:Coriobacteriaceae_UCG-002, g:Faecalibaculum, g:unclassified_f:Oscillospiraceae, g:norank_f:Oscillospiraceae, and g:norank_f:norank_o:norank_c:Clostridia. COG enrichment analysis of gut microbiota showed that cell wall/membrane/envelope biogenesis, amino acid transport and metabolism, translation/ribosomal structure and biogenesis, carbohydrate transport and metabolism, replication/recombination and repair, energy production and conversion, transcription, inorganic ion transport and metabolism, coenzyme transport and metabolism, nucleotide transport and metabolism, and lipid transport and metabolism were mainly involved in the control and dampness groups (**Figure 4D**).

### 3.5 Serum metabolism profile in mice with environmental high humidity

**Figure 5:**
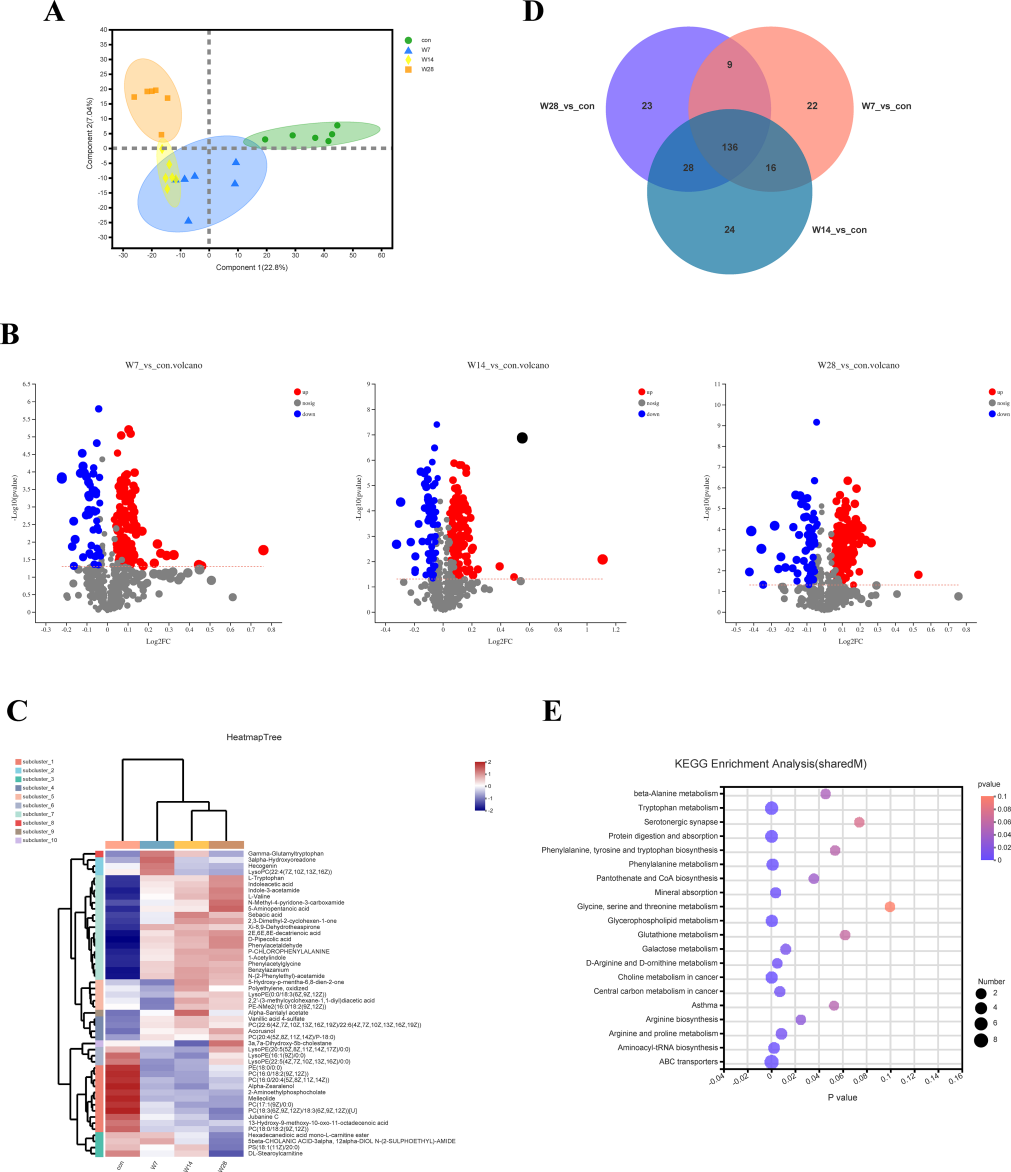
Serum metabolism profile in mice with environmental high humidity. **(A)** Score plots of partial least squares-discriminant analysis (PLS-DA). **(B)** Volcano plot of differential metabolites in the W7 vs. Con groups, W14 vs. Con groups, and W28 vs. Con groups. **(C)** Heatmap of the top 50 differential metabolites in four groups. **(D)** Venn analysis. **(E)** The bubble chart of the KEGG pathway of 136 shared differential metabolites in all three comparison groups. con, control group; w7, w14, and w28 indicate groups housed under high-humidity conditions for 7, 14, and 28 days, respectively.

Metabolites were detected in the serum samples from control and dampness mice by LC/MS. A PLS-DA score plot was generated, and the permutation test showed that the model was reliable without overfitting [*R*^2^ = (0.0, 0.6332), Q2 = (0.0, −0.7783)] in the discovery set. As shown in **Figure 5A**, the PLS-DA plot proved the apparent separation between the control and dampness groups, implying obvious changes in the serum metabolism profile in control mice with environmental high humidity. The volcano plots showed that 131, 137, and 130 metabolites, respectively, from the W7, W14, and W28 groups of dampness mice were significantly upregulated, while 52, 67, and 66 metabolites were significantly downregulated compared with the control mice (*p* < 0.05) (**Figure 5B**). The relative concentrations of the top 50 differential metabolites in the four groups were shown in the heatmaps (**Figure 5C**), which have clear clustering and separation. As shown in **Figure 5D** and **Supplementary Table 2**, 136 differential metabolites were shared in the W7 vs. Con groups, W14 vs. Con groups, and W28 vs. Con groups. Enrichment pathway analysis of 136 shared differential metabolites in all three comparison groups is shown in **Figure 5E**, implying that dampness-induced metabolic disturbances were mainly associated with lipid metabolism (e.g., glycerophospholipid metabolism), amino acid metabolism (e.g., arginine and proline metabolism, tryptophan metabolism, phenylalanine metabolism, arginine biosynthesis), metabolism of other amino acids (e.g., D-arginine and D-ornithine metabolism, beta-alanine metabolism), cancers: overview (e.g., choline metabolism in cancer, central carbon metabolism in cancer), digestive system (e.g., protein digestion and absorption, mineral absorption), translation (e.g., aminoacyl-tRNA biosynthesis), membrane transport (e.g., ABC transporters), metabolism of cofactors and vitamins (e.g., pantothenate and CoA biosynthesis), and carbohydrate metabolism (e.g., galactose metabolism).

### 3.6 Correlation between gut microbiota and serum metabolites in mice with environmental high humidity

**Figure 6:**
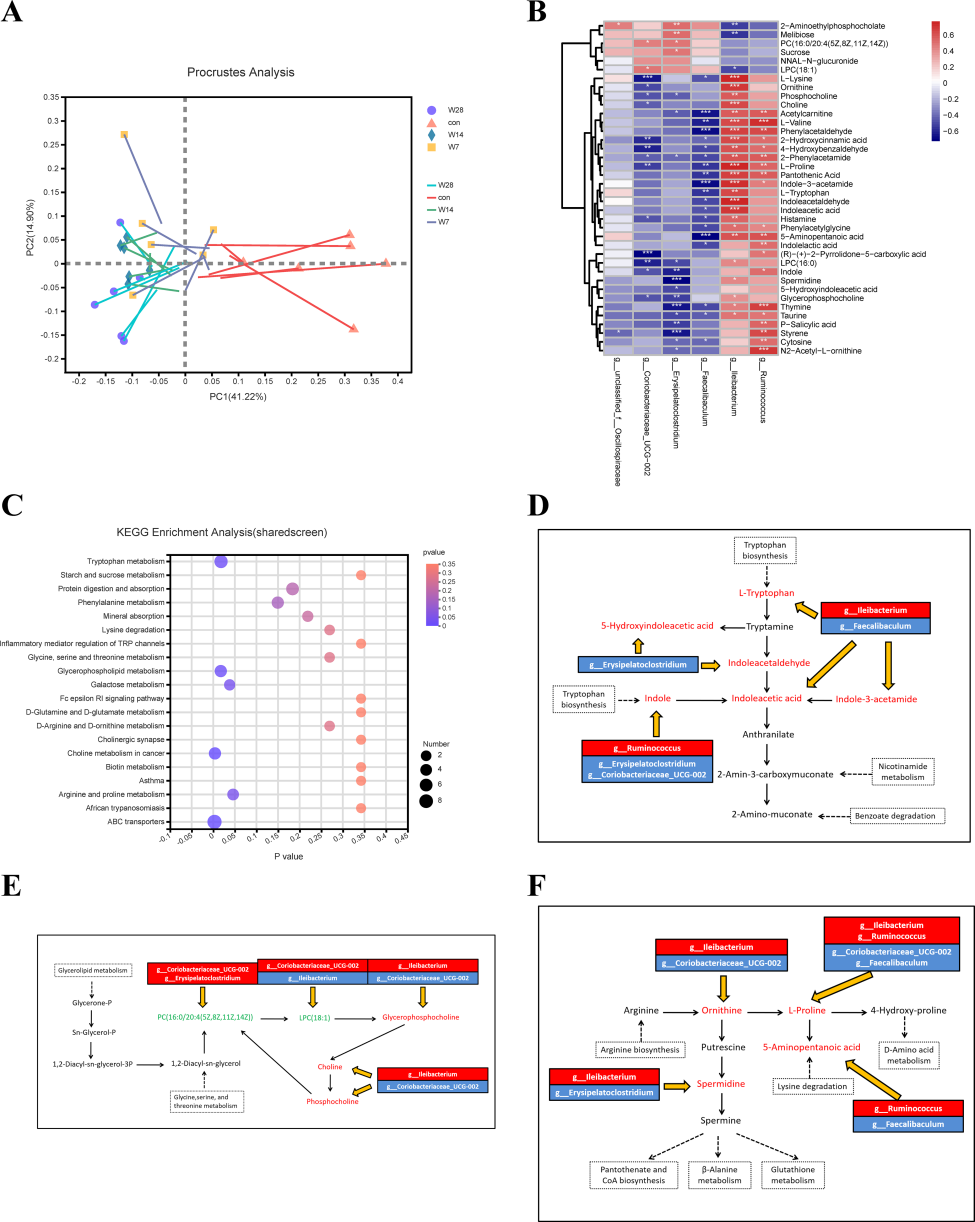
Correlation between gut microbiota and serum metabolites in mice with environmental high humidity. **(A)** Score plots of Procrustes analysis. **(B)** Correlation heatmap analysis between 6 intestinal flora and 38 microbiota-related metabolites screened based on *p <*0.05. **(C)** The bubble chart of the KEGG pathway in 38 microbiota-related metabolites. **(D)** Metabolic pathway map of tryptophan metabolism. **(E)** Metabolic pathway map of arginine and proline metabolism. **(F)** Metabolic pathway map of glycerophospholipid metabolism. The metabolites expressed in red indicate statistical upregulation, and those in green indicate statistical downregulation in the dampness group. Other related metabolic pathways were expressed in a dotted wire frame. The intestinal flora in red boxes were positively correlated, while those in blue boxes were negatively correlated with the indicated metabolites. *p < 0.05, **p < 0.01, ***p < 0.001. con, control group; w7, w14, and w28 indicate groups housed under high-humidity conditions for 7, 14, and 28 days, respectively.

Interomics correlation analyses were used to further explore the correlation between the gut microbiota and serum metabolome. Procrustes analysis showed that the trend of microbiome abundance and metabolomics expression was significantly consistent between the control and dampness groups (*p* = 0.039) (**Figure 6A**). A correlation between 6 intestinal flora and 136 shared different serum metabolites was calculated, among which 38 microbiota-related metabolites were screened based on *p <*0.05 and illustrated in a heatmap (**Figure 6B**). The results of the metabolic pathway enrichment analysis indicated that there were six enriched pathways with significant differences between the control and dampness groups, including tryptophan metabolism, glycerophospholipid metabolism, galactose metabolism, choline metabolism in cancer, arginine and proline metabolism, and ABC transporters (**Figure 6C**). The pathway networks between the intestinal flora and microbiota-related metabolites enriched in arginine and proline metabolism, glycerophospholipid metabolism, and tryptophan metabolism were constructed. The levels of microbiota-related metabolites enriched in these three pathways are shown in **Supplementary Figure 4**.

As shown in **Figure 6D**, six microbiota-related metabolites enriched in the tryptophan metabolic pathway (L-tryptophan, indole, indole-3-acetamide, indoleacetaldehyde, indoleacetic acid, and 5-hydroxyindoleacetic acid) had higher concentrations in the dampness group compared with the control group. Among the six intestinal flora, g:Ileibacterium was positively correlated and g:Faecalibaculum was negatively correlated with L-tryptophan, indole-3-acetamide, and indoleacetic acid. g:Erysipelatoclostridium was negatively correlated with indole, indoleacetaldehyde, and 5-hydroxyindoleacetic acid, while g:Ruminococcus was positively correlated with indole.

On the arginine and proline metabolic pathway (**Figure 6E**), the enriched microbiota-related metabolites were L-proline, ornithine, 5-aminopentanoic acid, and spermidine, highly expressed in the dampness group when compared with the control group. Among the six intestinal flora, g:Ileibacterium was positively correlated with L-proline, ornithine, and spermidine, and g:Ruminococcus was positively correlated with L-proline and 5-aminopentanoic acid. In addition, g:Coriobacteriaceae_UCG-002 was negatively correlated with L-proline and ornithine, and g:Faecalibaculum was negatively correlated with L-proline and 5-aminopentanoic acid.

As microbiota-related metabolites enriched in the glycerophospholipid metabolic pathway (**Figure 6F**), phosphocholine, choline, and glycerophosphocholine were highly expressed, while LPC(18:1) and PC(16:0/20:4(5Z,8Z,11Z,14Z)) had low expression in the dampness group when compared with the control group. Among the six intestinal flora, g:Coriobacteriaceae_UCG-002 was positively correlated with LPC(18:1) and PC(16:0/20:4(5Z,8Z,11Z,14Z)), while it was negatively correlated with phosphocholine, choline, and glycerophosphocholine. g:Ileibacterium was positively correlated with phosphocholine, choline, and glycerophosphocholine, while it was negatively correlated with LPC(18:1). In addition, g:Erysipelatoclostridium was positively correlated with PC(16:0/20:4(5Z,8Z,11Z,14Z)).

### 3.7 Gut microbiota-related metabolites shared in rats and mice with environmental high humidity

**Figure 7:**
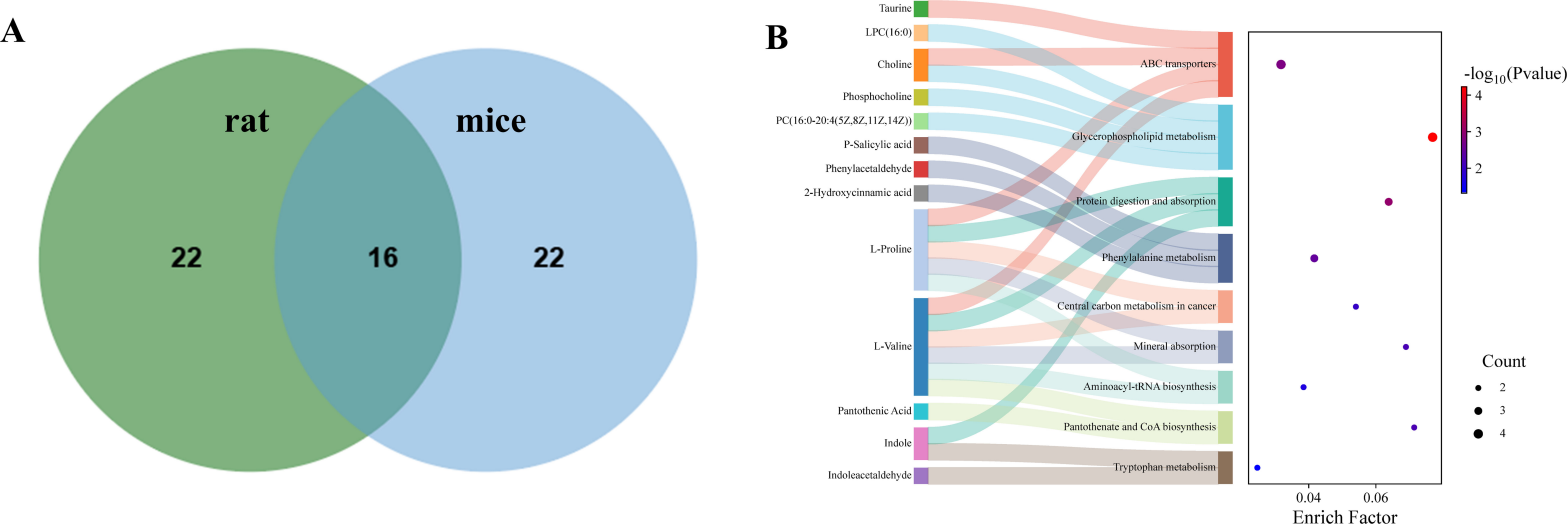
Gut microbiota-related metabolites shared in rats and mice with environmental high humidity. **(A)** Venn analysis presented the common metabolites between rats and mice. **(B)** Sankey plots showed the KEGG enrichment (significant metabolic pathways with *p* < 0.05) on shared metabolites.

As shown in **Figure 7A**, there were 16 gut microbiota-related metabolites shared in rats and mice under high-humidity exposure. A Sankey plot was generated to show the KEGG enrichment (significant metabolic pathways with *p* < 0.05) on these shared metabolites. As shown in **Figure 7B**, the shared metabolites, including phosphocholine, choline, LPC (16:0), taurine, L-valine, L-proline, 2-hydroxycinnamic acid, phenylacetaldehyde, P-salicylic acid, and PC(16:0/20:4(5Z,8Z,11Z,14Z)), were significantly enriched in glycerophospholipid metabolism, ABC transporters, and phenylalanine metabolism. These results suggested that cells may synthesize essential metabolic products that potentially construct and maintain functional membrane structures, and could further participate in regulating the transport of substances across the membrane, thereby plausibly helping to maintain intracellular homeostasis in response to high-humidity exposure.

## 4 Discussion

In this study, by establishing animal models of rats and mice subjected to prolonged exposure to a high-humidity environment, combined with 16S rRNA sequencing and LC-MS non-targeted metabolomics technology, we systematically revealed the regulatory effect of high humidity on the host’s intestinal microecology and metabolic homeostasis. The regulatory axis of “high humidity–gut microbiota dysbiosis–metabolic disorder” shows both commonalities and specificities between rats and mice.

The stability of the gut microbiota is an important guarantee for the health of the host, and its structural changes are closely related to environmental exposure (Koziol et al., 2023). Although high-humidity exposure did not significantly alter the α diversity of the gut microbiota in rats and mice, β diversity analysis of our study revealed a significant separation at the OTU level between the high-humidity group and the control group, suggesting that high-humidity exposure mainly affects the composition of the microbiota rather than species abundance. This finding is consistent with the previous report about the regulatory characteristics of environmental stress on microbial communities (Lei et al., 2025). At the phylum level, the dominant phyla in both models were Bacteroidetes and Firmicutes, which conformed to the typical composition characteristics of the mammal intestinal microbiota (Fu et al., 2025). However, the effect of high humidity on the ratio of Firmicutes to Bacteroidetes (F/B ratio) showed differences between rats and mice. The F/B ratio decreased in hyperhumidity-exposed rats but increased in mice. This ratio is linked to metabolic phenotype and metabolic disorder risk (Wang et al., 2023), and its species-specific fluctuation may reflect differential metabolic adaptation to environmental exposure (Gao et al., 2023). Given the absence of significant changes in body weight, food intake, or water consumption in either species, this divergence likely arises from species-specific differences in energy metabolism and thermoregulation. At standard housing temperatures (22°C), mice are below thermoneutrality and experience chronic mild cold stress, which elevates basal metabolic rate, whereas rats, with a larger body size and lower surface-to-volume ratio, have a milder cold burden and more stable energy expenditure. High humidity impairs insulation and evaporative cooling, likely exacerbating thermal stress. In mice already operating at elevated metabolic cost, this may further increase energy demand, driving a compensatory gut microbiota shift toward a higher F/B ratio to enhance energy harvest. In rats, high humidity may act as a direct stressor without imposing major additional energy requirements, leading to dysbiosis reflected in a reduced F/B ratio. These findings highlight the need to account for species-specific physiological baselines when interpreting microbiota changes under environmental stress. Moreover, the divergent rat and mouse responses may offer complementary insights into how humans with distinct metabolic phenotypes respond to humid environments, thus reinforcing the translational value of these models.

In addition to species composition, LEfSe analysis identified the enriched genera related to high humidity in different groups of rats and mice. *Helicobacter*, one of the genera enriched in both rats and mice, has been reported to include strains that can induce intestinal inflammation and damage the integrity of the mucosal barrier by activating the TLR4/NF-κB signaling pathway (Gao et al., 2023). *Allobaculum* was significantly enriched in rats, while *Weissella* was significantly enriched in mice under high-humidity exposure of 7, 14, and 28 days. The changes in the relative abundance of *Allobaculum* were directly related to the remodeling of lipid metabolism in the host (Cao et al., 2025). Short-chain fatty acids produced by *Weissella*, a key energy source for intestinal epithelial cells, have been reported to affect intestinal barrier function and electrolyte and water absorption (Devi et al., 2021; Mann et al., 2024). COG functional enrichment analysis showed that the microbiota in the high-humidity groups of both mice and rats were mainly enriched in metabolic functions, implying that the high-humidity exposure-induced alteration of gut microbiota might be related to its regulation of metabolism. However, this approach is limited to functional inference, as the COG database assigns genes only to broad functional categories rather than specific metabolic pathways. The present findings, therefore, remain preliminary, and metagenomic or metatranscriptomic approaches are needed for validation. Moreover, the observed gut microbiome shifts may reflect not only host physiological responses but also direct environmental microbial seeding. Given that relative humidity strongly shapes the built environment microbiome (Chen et al., 2023; Fu et al., 2020), the 90% RH maintained in our chambers likely altered the microbial composition of bedding and air. Frequent rodent grooming and bedding contact could then facilitate direct microbial inoculation into the gut. Without sequencing of chamber surfaces or bedding, host-mediated effects cannot be disentangled from environmental transmission. This limitation should be considered when interpreting the gut microbiota changes, which may arise from both host and environmental sources. Future studies should address this gap by systematic environmental sampling (e.g., surface and air swabbing with 16S rRNA gene sequencing) to enable rigorous source attribution.

Previous reports have indicated that dysbiosis of the microbiota may cause its derived metabolites to enter the bloodstream, thereby forming specific metabolite characteristics in the serum (Fu et al., 2023). Therefore, we simultaneously observed changes in the host’s serum metabolites using serum non-target metabolomics. The results of the metabolomics analysis revealed that high-humidity exposure significantly altered the serum metabolic profiles of both rats and mice. PCA analysis showed that the differential metabolites were significantly clustered between groups, suggesting differences in the metabolic profiles between exposure to a normal and a high-humidity environment. To further reveal the relationship between microbiota changes and metabolite alterations, Pearson correlation was used to generate a correlation heatmap between the differential microbes and metabolites in our study. Procrustes analysis confirmed that the compositions of the gut microbiota in the models of rats and mice were highly consistent with the changing trend of the serum metabolic profile (*p* = 0.047, *p* = 0.039), suggesting that the gut microbiota may be an important mediator of metabolic disorders induced by high humidity. The correlation heatmap shows that multiple differential genera are significantly correlated with metabolites from the core metabolic pathway. UCG-005 was positively correlated with uracine and L-histidine, while Eubacterium_siraeum_group was negatively correlated with pantothenic acid and L-valine in rats exposed to high humidity. *Ileibacterium* was positively correlated with L-tryptophan and indole derivatives, while Faecalibaculum was negatively correlated with L-tryptophan and L-proline in mice exposed to high humidity. Most of these bacterial genera are intestinal symbiotic bacteria, and their abundance changes can directly regulate the synthesis and transformation of metabolites. Muribaculaceae participates in carbohydrate metabolism and bile acid conversion by secreting β-glucosidase (Zhu et al., 2024), and a decrease in its abundance can lead to an imbalance in the bile acid pool (Yang et al., 2024). The continuous enrichment of *Ileibacterium* in the hyperhumidity group of mice may promote the catabolism of tryptophan by activating tryptophan oxygenase, resulting in the generation of excessive indole derivatives (Qian et al., 2024; Yong et al., 2024).

Though the functional enrichment of differential metabolites under high-humidity exposure in rats and mice showed different characteristics, there were some shared pathways, including glycerophospholipid metabolism, ABC transporters, and phenylalanine metabolism. These pathways are closely related to cell membrane stability (Teo et al., 2025), substance transport (Madry et al., 2025), and signal transduction (Luo et al., 2025; Zhang et al., 2025). These pathway alterations may represent common features of metabolic imbalance induced by high-humidity exposure.

Glycerophospholipids are the main components of biological membranes. Metabolic disorders of glycerophospholipids can directly damage the integrity of cell membranes and affect the synthesis of signal molecules (Lee et al., 2024). In this study, rats and mice exposed to high humidity showed abnormal glycerol phospholipid metabolism. In particular, choline and phosphocholine increased, while PCs (phosphatidylcholine) and LysoPCs (lysopCs) decreased in the hyperhumidity group of rats; phosphocholine, choline, and glycerophosphocholine increased, while LPC (18:1) and PC (16:0/20:4) decreased in the hyperhumidity group of mice. This consistency change suggests that hyperhumidity may impede the synthesis and transformation of glycerophospholipids by inhibiting the activity of phosphatidylcholine transferase (Roberts et al., 2025), thereby disrupting the stability of cell membranes and signal transduction, which is consistent with the previous report on the characteristics of heat stress metabolic disorders (Li et al., 2025). In addition, the ABC transport protein pathway is involved in the transmembrane transport of lipids, amino acids, and other substances. Studies have shown that dysfunction of the ABC transport protein pathway can lead to the accumulation of metabolic products and nutrient absorption disorders, further amplifying metabolic imbalance (Li et al., 2024). In our study, high-humidity exposure, as an important environmental stress factor, may interfere with the normal function of ABC transporters, resulting in exacerbating the abnormal accumulation of metabolic products and nutritional absorption disorders, thereby triggering or accelerating the vicious cycle of metabolic imbalance.

Abnormal metabolism of phenylalanine is another significant feature of rats and mice exposed to high humidity. Phenylalanine, as an essential aromatic amino acid, is a key precursor for the synthesis of tyrosine and downstream catecholamine neurotransmitters and thyroid hormones (Buchmueller et al., 2024). The disturbance of its metabolic pathways may indicate that the animal body is mobilizing the neuroendocrine system to regulate the basal metabolic rate, water–salt balance, and stress response in response to the damp and hot environment (Weng et al., 2024). It is worth noting that there is a close interaction between changes in the structure of the intestinal microbiota (such as the enrichment of *Weissella* and the production of short-chain fatty acids) and the amino acid metabolism of the host. Microbial metabolites can act as signaling molecules influencing host metabolism, while host metabolic and stress status can reshape the microbiota (Xiong et al., 2025; Yao et al., 2025). Consistent with this, we observed enrichment of *Weissella*, suggesting that high-humidity stress may influence neuroendocrine and metabolic regulation via the microbiota–gut–brain axis, though this requires mechanistic validation. The aberrant phenylalanine metabolism may thus reflect part of a broader interplay among environmental stress, microbiota dysbiosis, and host metabolic–neuroendocrine responses.

## 5 Conclusion

The present study highlighted that high-humidity exposure disrupts the host’s metabolic homeostasis by altering the gut microbiota-related metabolites in rat and mouse models, showing commonalities and specificities. Our findings revealed that disorders in glycerophospholipid metabolism, ABC transporters, and amino acid metabolism as key metabolic characteristics of hyperhumidity exposure may provide new ideas and insights for further study on the pathogenic mechanism of hyperhumidity. However, this study has some limitations. The detection of intestinal histology and functional enzyme activity is lacking in this study. The causal role of the intestinal microbiota needs to be clarified through experimental verification, such as fecal transplantation.

## Data Availability

The datasets presented in this study can be found in online repositories. The names of the repository/repositories and accession number(s) can be found below: https://www.ncbi.nlm.nih.gov/, PRJNA868863 https://www.ncbi.nlm.nih.gov/, PRJNA867526 https://www.ebi.ac.uk/metabolights/, MTBLS5660 https://www.ebi.ac.uk/metabolights/, MTBLS5653.

## References

[B1] Buchmueller L. C. Wunderle C. Laager R. Bernasconi L. Neyer P. J. Tribolet P. . (2024). Association of phenylalanine and tyrosine metabolism with mortality and response to nutritional support among patients at nutritional risk: a secondary analysis of the randomized clinical trial EFFORT. Front. Nutr. 11, 1451081. doi: 10.3389/fnut.2024.1451081 39600719 PMC11588475

[B2] Cao X. Wang J. Zhang Y. Zhang J. Li J. (2025). Association of gut microbiota and arginine-agmatine metabolism with postoperative delirium in elderly patients following lower limb fracture surgery: a prospective nested case-control study. Arch. Gerontol. Geriatr. 142, 106094. doi: 10.1016/j.archger.2025.106094 41314165

[B3] Chen Y. Fu X. Ou Z. Li J. Lin S. Wu Y. . (2023). Environmental determinants and demographic influences on global urban microbiomes, antimicrobial resistance and pathogenicity. NPJ Biofilms Microbomes 9, 94. doi: 10.1038/s41522-023-00459-4 38062054 PMC10703778

[B4] Chen S. Liu C. Lin G. Hänninen O. Dong H. Xiong K. (2021). The role of absolute humidity in respiratory mortality in guangzhou, a hot and wet city of south China. Environ. Health Prev. 26, 109. doi: 10.1186/s12199-021-01030-3 34789160 PMC8597241

[B5] Devi P. B. Kavitake D. Jayamanohar J. Shetty P. H. (2021). Preferential growth stimulation of probiotic bacteria by galactan exopolysaccharide from weissella confusa KR780676. Food. Res. Int. (Ottawa Ont.) 143, 110333. doi: 10.1016/j.foodres.2021.110333 33992335

[B6] Dimitrova A. Ingole V. Basagaña X. Ranzani O. Milà C. Ballester J. . (2021). Association between ambient temperature and heat waves with mortality in south asia: systematic review and meta-analysis. Environ. Int. 146, 106170. doi: 10.1016/j.envint.2020.106170 33395923

[B7] Freire-Zapata V. Holland-Moritz H. Cronin D. R. Aroney S. Smith D. A. Wilson R. M. . (2024). Microbiome–metabolite linkages drive greenhouse gas dynamics over a permafrost thaw gradient. Nat. Microbiol. 9, 2892–2908. doi: 10.1038/s41564-024-01800-z 39354152 PMC11522005

[B8] Fu X. Li Y. Yuan Q. Cai G. Deng Y. Zhang X. . (2020). Continental-scale microbiome study reveals different environmental characteristics determining microbial richness, composition, and quantity in hotel rooms. Msystems 5, e00119-e00120. doi: 10.1128/mSystems.00119-20 32430405 PMC7253364

[B9] Fu J. Shan J. Cui Y. Yan C. Wang Q. Han J. . (2023). Metabolic disorder and intestinal microflora dysbiosis in chronic inflammatory demyelinating polyradiculoneuropathy. Cell Biosci. 13, 6. doi: 10.1186/s13578-023-00956-1 36627678 PMC9832664

[B10] Fu Z. Zhang H. Yang Z. Liu Y. Wang P. Zhang J. . (2025). Metagenomic and metabolomic analyses reveal the role of a bacteriocin-producing strain of enterococcus faecalis DH9003 in regulating gut microbiota in mice. Microorganisms 13, 372. doi: 10.3390/microorganisms13020372 40005739 PMC11858018

[B11] Gao H. Jiang F. Zhang J. Chi X. Song P. Li B. . (2023). Effects of ex situ conservation on diversity and function of the gut microbiota of the tibetan wild ass (equus kiang). Integr. Zool. 18, 1089–1104. doi: 10.1111/1749-4877.12726 37231976

[B12] Koziol A. Odriozola I. Leonard A. Eisenhofer R. San José C. Aizpurua O. . (2023). Mammals show distinct functional gut microbiome dynamics to identical series of environmental stressors. Mbio 14, e0160623. doi: 10.1128/mbio.01606-23 37650630 PMC10653949

[B13] Kvitne K. E. Zuffa S. Charron-Lamoureux V. Mohanty I. Patan A. Mannochio-Russo H. . (2026). Fecal microbial and metabolic signatures in children with very early onset inflammatory bowel disease. NPJ Biofilms Microbomes 12, 33. doi: 10.1038/s41522-025-00899-0 41476253 PMC12864830

[B14] Lee R. G. Rudler D. L. Rackham O. Filipovska A. (2024). Interorganelle phospholipid communication, a house not so divided. Trends Endocrinol. Metabolism: TEM 35, 872–883. doi: 10.1016/j.tem.2024.06.008 38972781

[B15] Lei C. Zhou S. Tissue D. T. Neilson R. Lie Z. Wu T. . (2025). Seasonal variation of phyllosphere microbial communities under warming. Glob. Change Biol. 31, e70270. doi: 10.1111/gcb.70270 40452412

[B16] Li Y. Chen Z. Xu D. Wang L. Cheng M. Zhou C. . (2024). Structural insights into human ABCD3-mediated peroxisomal acyl-CoA translocation. Cell. Discov. 10, 92. doi: 10.1038/s41421-024-00722-8 39223112 PMC11369193

[B17] Li Q. Zhou X. Zhang X. Zhang C. Zhang S. O. (2025). Nuclear receptor signaling regulates compartmentalized phosphatidylcholine remodeling to facilitate thermosensitive lipid droplet fusion. Nat. Commun. 16, 3955. doi: 10.1038/s41467-025-59256-6 40289189 PMC12034805

[B18] Luo P. Tong K. Gan Y. Tang M. Niu Y. Liu K. . (2025). Amino acid-sensing neurons in the anterior piriform cortex control brown adipose tissue thermogenesis. Adv. Sci. 12, e2502421. doi: 10.1002/advs.202502421 40305738 PMC12279160

[B19] Madry C. Elbahnsi A. Delaunay J. Stary A. Lagaye S. Couvert P. . (2025). ABCB4 disease-causing variants s242r, s346i, t437i and t1077m significantly impair its function and display differential sensitivity to potentiators. Sci. Rep. 15, 44544. doi: 10.1038/s41598-025-28407-6 41274965 PMC12739136

[B20] Mann E. R. Lam Y. K. Uhlig H. H. (2024). Short-chain fatty acids: linking diet, the microbiome and immunity. Nat. Rev. Immunol. 24, 577–595. doi: 10.1038/s41577-024-01014-8 38565643

[B21] Qian X. Li Q. Zhu H. Chen Y. Lin G. Zhang H. . (2024). Bifidobacteria with indole-3-lactic acid-producing capacity exhibit psychobiotic potential via reducing neuroinflammation. Cell Rep. Med. 5, 101798. doi: 10.1016/j.xcrm.2024.101798 39471819 PMC11604549

[B22] Rahman M. M. McKeon K. Luglio D. Hossain C. A. Rabito F. Alam N. . (2025). Compounding effects of heat and high humidity on cardiovascular morbidity in dhaka, Bangladesh: an implication of climate crisis. Sci. Total Environ. 995, 180220. doi: 10.1016/j.scitotenv.2025.180220 40813191

[B23] Roberts J. R. Horibata Y. Kwarcinski F. E. Lam V. Raczkowski A. M. Hubbard A. . (2025). Structural basis for catalysis and selectivity of phospholipid synthesis by eukaryotic choline-phosphotransferase. Nat. Commun. 16, 111. doi: 10.1038/s41467-024-55673-1 39747155 PMC11696302

[B24] Teo C. F. Tuomivaara S. T. van Hilten N. Crottès D. Jan Y. N. Grabe M. . (2025). A cell-based scrambling assay reveals the phospholipid headgroup preference of TMEM16f on the plasma membrane. Proc. Natl. Acad. Sci. U.S.A. 122, e2516822122. doi: 10.1073/pnas.2516822122 41166415 PMC12595458

[B25] Vecellio D. J. Kong Q. Kenney W. L. Huber M. (2023). Greatly enhanced risk to humans as a consequence of empirically determined lower moist heat stress tolerance. Proc. Natl. Acad. Sci. U.S.A. 120, e2305427120. doi: 10.1073/pnas.2305427120 37812703 PMC10589700

[B26] Wang J. Li N. Liu Y. Zheng H. Wu R. Zhang J. . (2026). Holstein cow milk-derived lactobacillus plantarum l19 alleviates heat stress-induced liver injury in mice by modulating gut microbiota. J. Dairy Sci. 109, 17–33. doi: 10.3168/jds.2025-26920 41274573

[B27] Wang W. Xia Y. Zhang P. Zhu M. Huang S. Sun X. . (2025). Narrow-spectrum resource-utilizing bacteria drive the stability of synthetic communities through enhancing metabolic interactions. Nat. Commun. 16, 6088. doi: 10.1038/s41467-025-61432-7 40603291 PMC12222865

[B28] Wang K. Zhao Y. Xu L. Liao X. Xu Z. (2023). Health outcomes of 100% orange juice and orange flavored beverage: a comparative analysis of gut microbiota and metabolomics in rats. Curr. Res. Food Sci. 6, 100454. doi: 10.1016/j.crfs.2023.100454 36815996 PMC9932342

[B29] Weng H. Deng L. Wang T. Xu H. Wu J. Zhou Q. . (2024). Humid heat environment causes anxiety-like disorder via impairing gut microbiota and bile acid metabolism in mice. Nat. Commun. 15, 5697. doi: 10.1038/s41467-024-49972-w 38972900 PMC11228019

[B30] Won T. H. Arifuzzaman M. Parkhurst C. N. Miranda I. C. Zhang B. Hu E. . (2025). Host metabolism balances microbial regulation of bile acid signalling. Nature 638, 216–224. doi: 10.1038/s41586-024-08379-9 39779854 PMC11886927

[B31] Wu Y. Feng X. Li M. Hu Z. Zheng Y. Chen S. . (2024). Gut microbiota associated with appetite suppression in high-temperature and high-humidity environments. EBioMedicine 99, 104918. doi: 10.1016/j.ebiom.2023.104918 38103514 PMC10765014

[B32] Xiong L. Diwakarla S. Chatzis R. Artaiz O. Macowan M. Zhang S. . (2025). Acute exposure to high-fat diet impairs ILC3 functions and gut homeostasis. Immunity 58, 1185–1200. doi: 10.1016/j.immuni.2025.03.017 40233759

[B33] Yang X. Xu Y. Li J. Ran X. Gu Z. Song L. . (2024). Bile acid-gut microbiota imbalance in cholestasis and its long-term effect in mice. Msystems 9, e0012724. doi: 10.1128/msystems.00127-24 38934542 PMC11265269

[B34] Yao Y. Pei Z. Dai Y. Chen Y. Chang Z. Wang H. . (2025). Akkermansia muciniphila-derived n-acetylspermidine modulates the localization of intestinal α1,2-fucosylated proteins to maintain gut homeostasis. Adv. Sci. 12, e06576. doi: 10.1002/advs.202506576 40776401 PMC12520552

[B35] Yong C. C. Sakurai T. Kaneko H. Horigome A. Mitsuyama E. Nakajima A. . (2024). Human gut-associated bifidobacterium species salvage exogenous indole, a uremic toxin precursor, to synthesize indole-3-lactic acid via tryptophan. Gut Microbes 16, 2347728. doi: 10.1080/19490976.2024.2347728 38706226 PMC11085991

[B36] Zhang D. Song S. Lin J. Ye T. Yang X. Jiang Q. . (2025). Glutamine binds HSC70 to transduce signals inhibiting IFN-β-mediated immunogenic cell death. Dev. Cell 60, 1958–1973. doi: 10.1016/j.devcel.2025.02.012 40086433

[B37] Zheng C. Wu J. Tang H. Wang X. Tian Y. Cao X. . (2024). Relationship of ambient humidity with cardiovascular diseases: a prospective study of 24,510 adults in a general population. Biomed. Environ. Sci. BES 37, 1352–1361. doi: 10.3967/bes2024.156 40383942

[B38] Zhou C. Yang S. Ka W. Gao P. Li Y. Long R. . (2022). Association of gut microbiota with metabolism in rainbow trout under acute heat stress. Front. Microbiol. 13, 846336. doi: 10.3389/fmicb.2022.846336 35432278 PMC9007319

[B39] Zhu Y. Chen B. Zhang X. Akbar M. T. Wu T. Zhang Y. . (2024). Exploration of the muribaculaceae family in the gut microbiota: diversity, metabolism, and function. Nutrients 16, 2660. doi: 10.3390/nu16162660 39203797 PMC11356848

