# Supplementary material for: Integrated analysis of fecal microbiome and serum metabolome reveals the profiling of gut microbiota-related metabolites in rats and mice subjected to prolonged exposure to a high-humidity environment
Source: Front Cell Infect Microbiol. 2026 Jun 22;16:1782615. doi: 10.3389/fcimb.2026.1782615 (PMC13333707; doi:10.3389/fcimb.2026.1782615)
Supplement: Supplementary file 1 [file DataSheet1.docx]

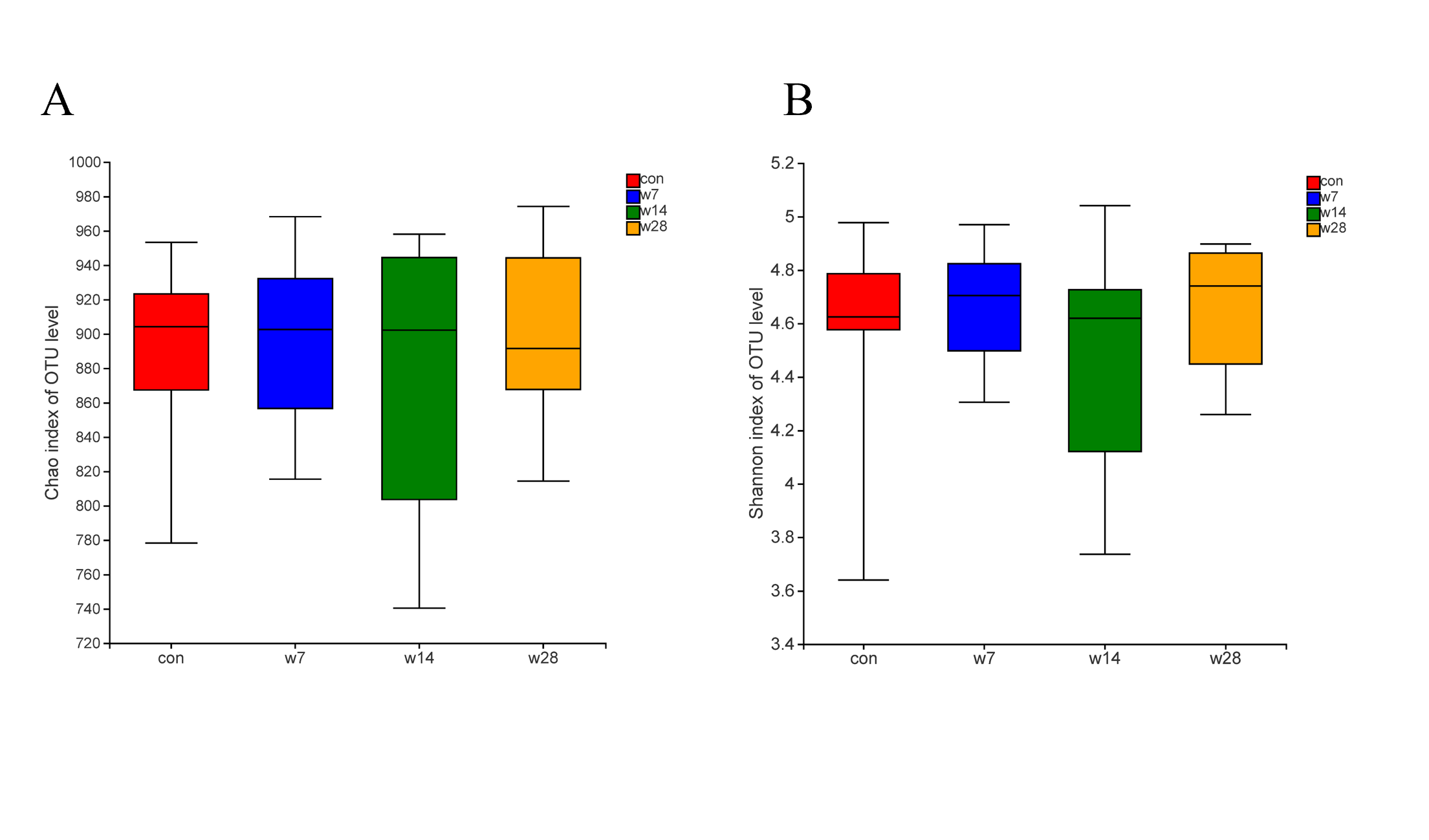


**Figure S1 Alpha diversity analysis in rats (n=6).** (A) Chao index of OUT level. (B)Shannon index of OUT level. con, control group; w7, w14, and w28 indicate groups housed under high-humidity conditions for 7, 14, and 28 days, respectively.





**Figure S2 Expression diagram of microbial-related metabolites in the biosynthesis of pantothenic acid and coenzyme A, β-alanine metabolism, and glycerophospholipid metabolism pathways of rats.** con, control group; w7, w14, and w28 indicate groups housed under high-humidity conditions for 7, 14, and 28 days, respectively.


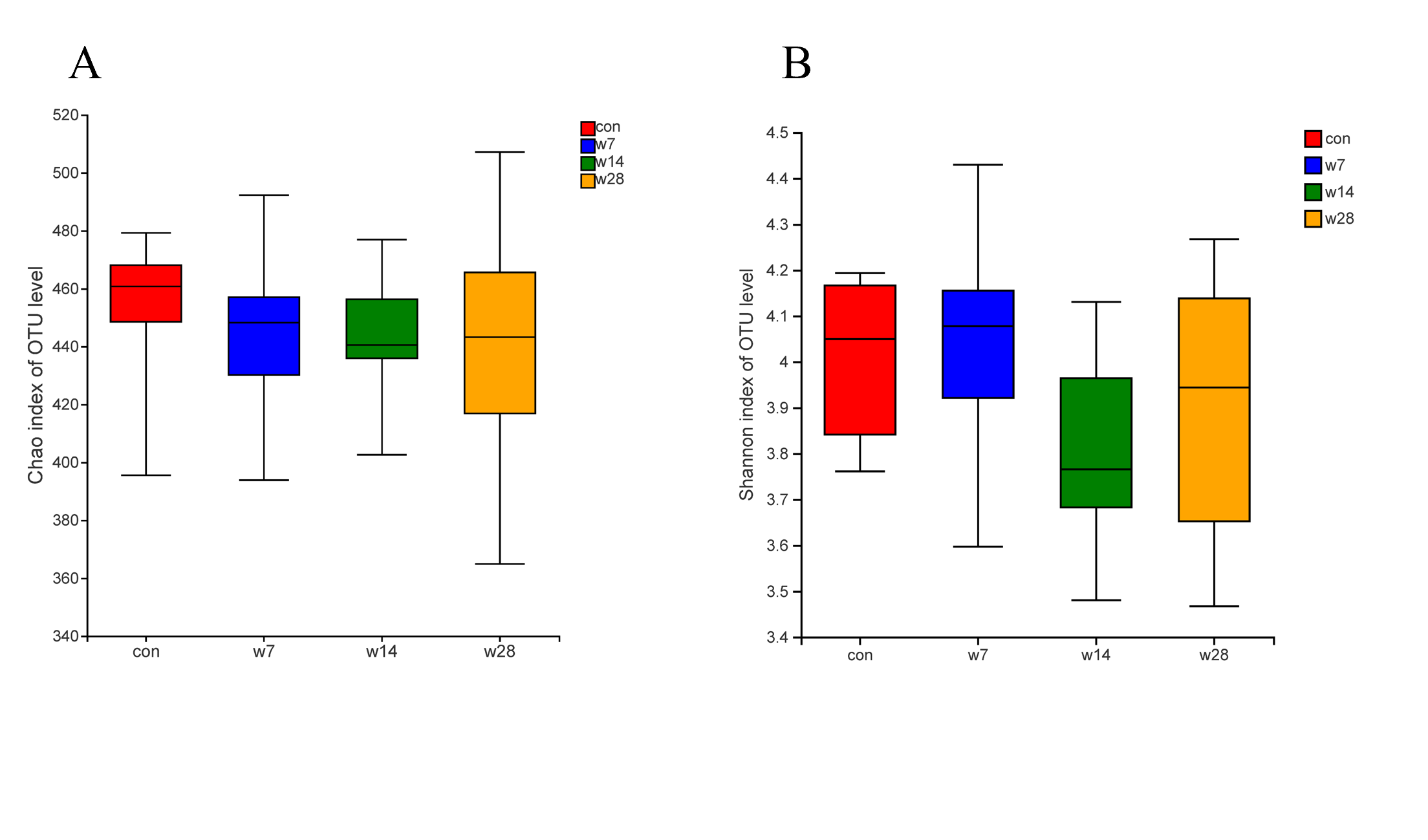


**Figure S3 Alpha diversity analysis in mice (n=6)**. (A) Chao index of OUT level. (B)Shannon index of OUT level. con, control group; w7, w14, and w28 indicate groups housed under high-humidity conditions for 7, 14, and 28 days, respectively.





**Figure S4 Expression diagram of microbial-related metabolites in the arginine and proline metabolism, glycerophospholipid metabolism, and tryptophan metabolism pathways of mice**. con, control group; w7, w14, and w28 indicate groups housed under high-humidity conditions for 7, 14, and 28 days, respectively.


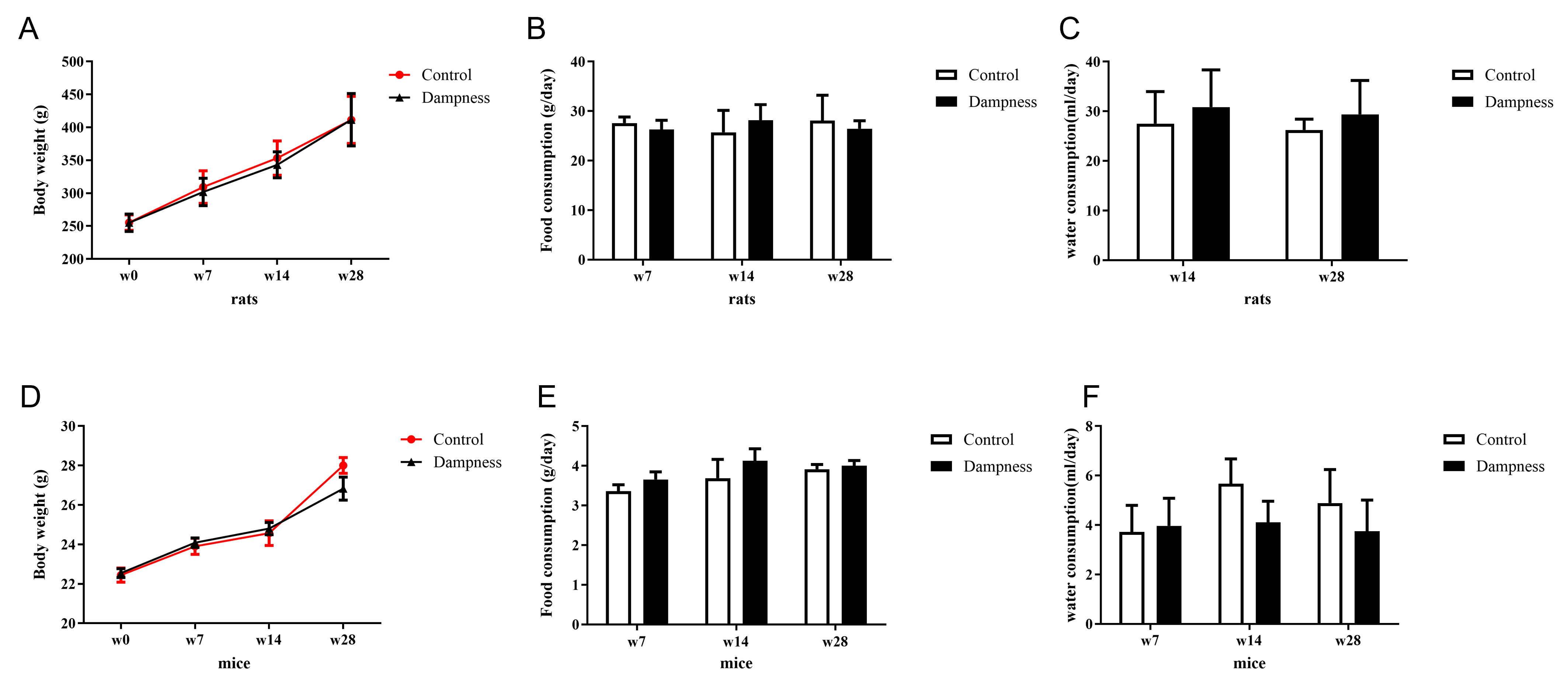


**Figure S5 General condition assessment of rats and mice (n=6) (A) Body weight change curves of rats, and (D) mice. (B) Quantification of food consumption in rats, and (E) mice. (C) Quantification of water consumption in rats, and (F) mice.** w7, w14, and w28 indicate groups housed under high-humidity conditions for 7, 14, and 28 days, respectively.


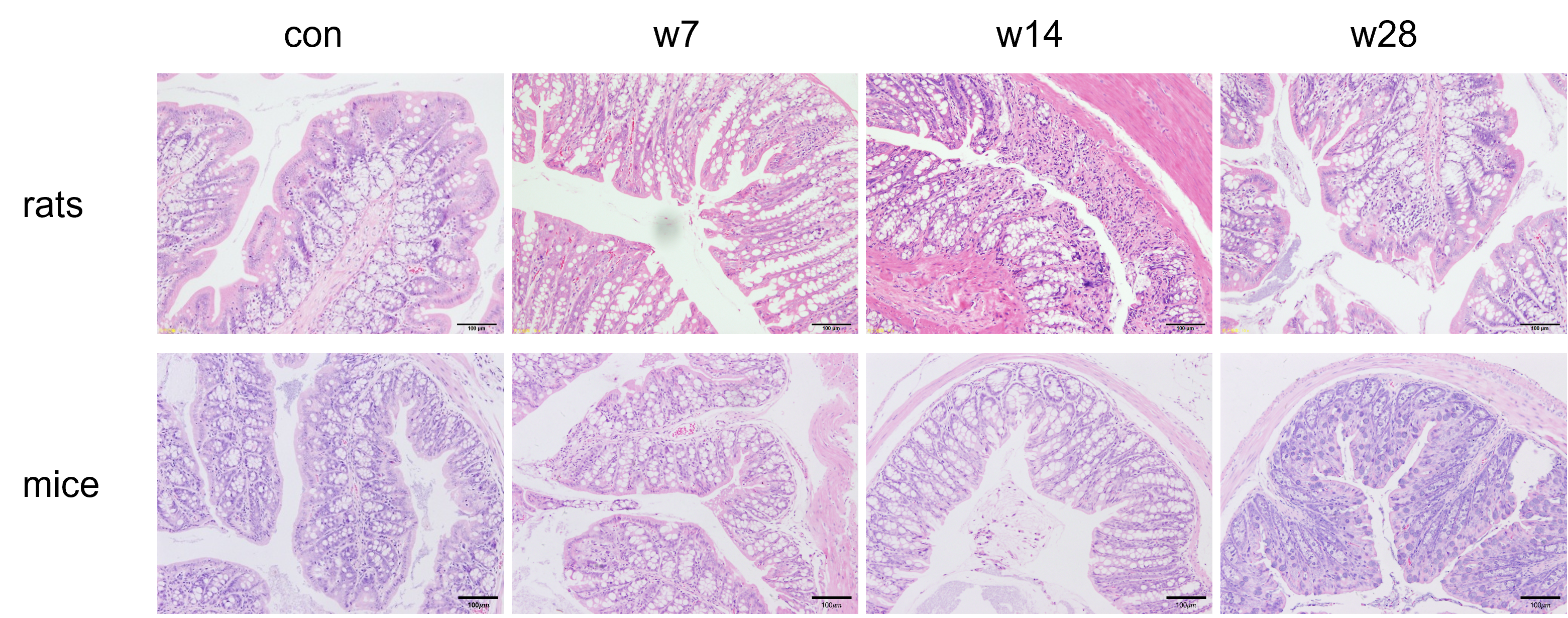


**Figure S6 HE staining of the colon tissues of rats and mice (n=6).** con, control group; w7, w14, and w28 indicate groups housed under high-humidity conditions for 7, 14, and 28 days, respectively.


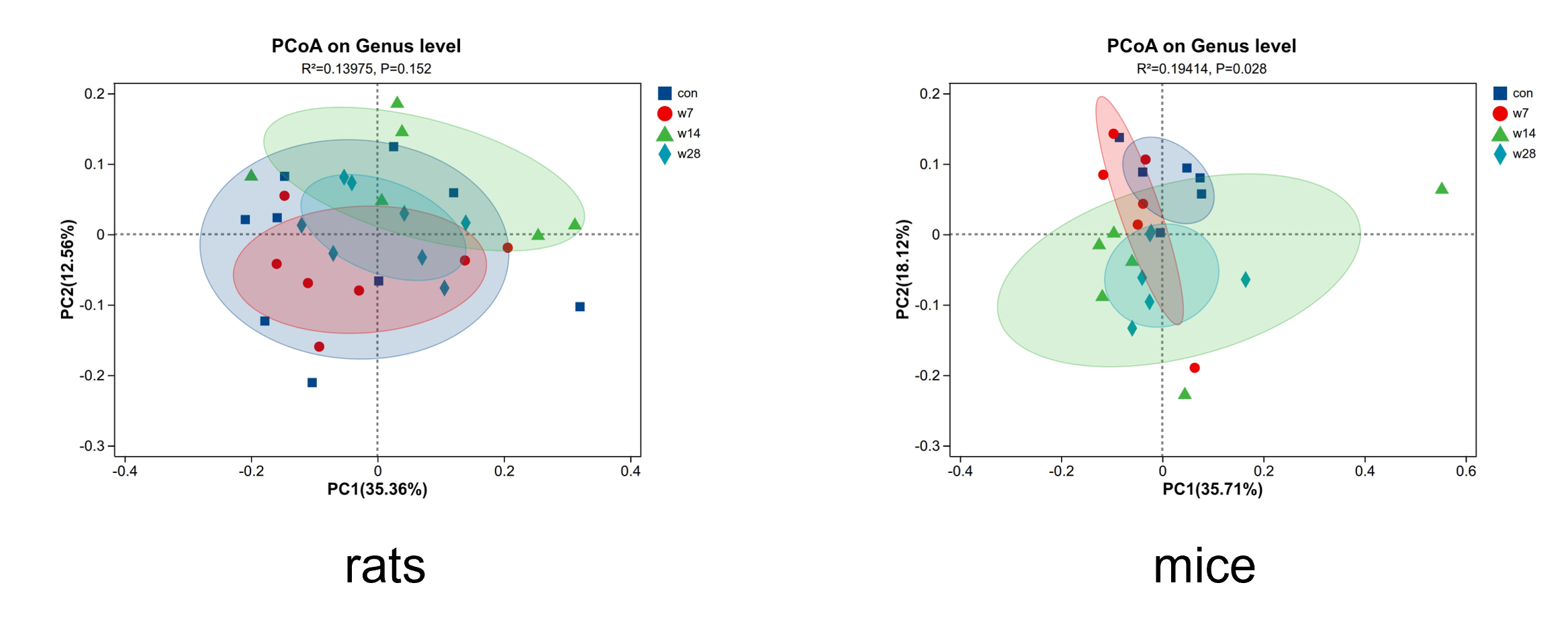


**Figure S7 Principal Coordinates Analysis (PCoA) of gut microbiota in rats and mice.(n=6)**
