# Supplementary material for: Integrated analysis of fecal microbiome and serum metabolome reveals the profiling of gut microbiota-related metabolites in rats and mice subjected to prolonged exposure to a high-humidity environment
Source: Front Cell Infect Microbiol. 2026 Jun 22;16:1782615. doi: 10.3389/fcimb.2026.1782615 (PMC13333707; doi:10.3389/fcimb.2026.1782615)
Supplement: Supplementary file 2 [file Table1.docx]

Table S1 The top 50 differential metabolites in rat serum between W7 and Control group.

| Metabolite | VIP_pred_OPLS-DA | VIP_PLS-DA | FC(W7/con) | P_value |
| --- | --- | --- | --- | --- |
| PC(15:0/20:2(11Z,14Z)) | 2.1555 | 2.0315 | 0.8947 | 4.37E-06 |
| Pantothenic Acid | 1.7041 | 1.5965 | 1.0561 | 5.11E-06 |
| Taurine | 1.911 | 1.7842 | 1.0853 | 7.66E-06 |
| PC(18:1(11Z)/22:6(4Z,7Z,10Z,13Z,16Z,19Z)) | 2.1256 | 1.9868 | 0.909 | 1.04E-05 |
| Phosphocholine | 1.8302 | 1.7082 | 1.0704 | 1.12E-05 |
| PC(15:0/18:2(9Z,12Z)) | 1.8911 | 1.7683 | 0.9208 | 2.33E-05 |
| Diplosporin | 1.3253 | 1.2337 | 1.0452 | 4.87E-05 |
| PC(19:1(9Z)/0:0) | 1.8339 | 1.7143 | 0.925 | 4.89E-05 |
| Tridecanoylglycine | 1.5121 | 1.4087 | 1.0629 | 5.74E-05 |
| PC(18:0/18:2(9Z,12Z)) | 1.7964 | 1.6833 | 0.9417 | 6.54E-05 |
| PC(14:0/18:2(9Z,12Z)) | 1.9543 | 1.8236 | 0.9179 | 7.22E-05 |
| 4-Chlorobenzaldehyde | 1.5479 | 1.4453 | 1.0515 | 7.78E-05 |
| PC(16:0/18:2(9Z,12Z)) | 1.637 | 1.5262 | 0.9559 | 7.98E-05 |
| Armexifolin | 2.9028 | 2.7003 | 1.3061 | 8.17E-05 |
| N-(3-acetamidopropyl)pyrrolidin-2-one | 1.9549 | 1.8283 | 1.0927 | 8.48E-05 |
| 6-Methoxymellein | 1.5052 | 1.4023 | 1.0602 | 9.43E-05 |
| N,N'-Dicyclohexylurea | 1.7308 | 1.61 | 1.1299 | 9.78E-05 |
| Dihydrocoumarin | 1.4538 | 1.3551 | 1.0397 | 9.91E-05 |
| S-Cysteinosuccinic acid | 1.3429 | 1.2559 | 0.9587 | 9.96E-05 |
| Benzaldehyde | 1.6781 | 1.5635 | 1.0777 | 0.000106 |
| Mangalkanyl glucoside | 1.5224 | 1.4325 | 0.9255 | 0.000109 |
| (S)-(-)-Perillyl alcohol | 1.6206 | 1.5105 | 1.0736 | 0.000127 |
| Methyl isobutyl ketone | 1.2518 | 1.1708 | 0.946 | 0.000138 |
| Indole | 1.5857 | 1.484 | 1.0468 | 0.000139 |
| 4-HYDROXY-6-METHYLPYRAN-2-ONE | 1.5892 | 1.4798 | 1.0648 | 0.00014 |
| 4-Phenyl-2-butanol | 1.6602 | 1.5505 | 1.0716 | 0.00014 |
| PC(16:0/18:3(6Z,9Z,12Z)) | 1.9037 | 1.7744 | 0.9285 | 0.000159 |
| LysoPC(16:0) | 1.7854 | 1.6685 | 0.9258 | 0.000176 |
| LysoPC(24:1(15Z)) | 1.809 | 1.6775 | 0.9208 | 0.000193 |
| Benzylazanium | 1.4445 | 1.3468 | 1.0412 | 0.000195 |
| PC(16:1(9Z)/22:6(4Z,7Z,10Z,13Z,16Z,19Z)) | 2.191 | 2.0474 | 0.8857 | 0.000196 |
| 4-Acetamidobutanoate | 1.832 | 1.6999 | 1.0881 | 0.000197 |
| N-(2-Phenylethyl)-acetamide | 1.5888 | 1.4842 | 1.0732 | 0.000198 |
| PE(14:1(9Z)/20:0) | 1.6283 | 1.5209 | 0.949 | 0.000209 |
| P-Salicylic acid | 1.5078 | 1.4084 | 1.0763 | 0.00021 |
| 2,6,10,10-Tetramethyl-1-oxaspiro[4.5]decan-6-ol | 1.3571 | 1.2676 | 1.0421 | 0.000219 |
| PC(16:0/22:4(7Z,10Z,13Z,16Z)) | 1.702 | 1.5871 | 0.9411 | 0.00024 |
| 4-Phenyl-3-buten-2-ol | 1.9341 | 1.8017 | 1.1006 | 0.00026 |
| Thymine | 1.6863 | 1.5771 | 1.081 | 0.000275 |
| PE-NMe2(22:5(4Z,7Z,10Z,13Z,16Z)/24:1(15Z)) | 1.8293 | 1.7111 | 0.9234 | 0.000277 |
| Alpha-Bisabolol oxide C | 1.0479 | 0.9782 | 1.0261 | 0.000346 |
| Phenylacetaldehyde | 1.7337 | 1.62 | 1.0877 | 0.000365 |
| PE(22:5(4Z,7Z,10Z,13Z,16Z)/22:6(4Z,7Z,10Z,13Z,16Z,19Z)) | 1.7078 | 1.6002 | 0.9493 | 0.000373 |
| Galegine | 1.3813 | 1.291 | 1.0589 | 0.000392 |
| Osmundalactone | 1.5104 | 1.3994 | 1.0574 | 0.000393 |
| PC(15:0/22:4(7Z,10Z,13Z,16Z)) | 1.9706 | 1.837 | 0.9157 | 0.000393 |
| 4-formyl Indole | 1.4868 | 1.4007 | 1.0464 | 0.000412 |
| Betaine aldehyde | 1.4996 | 1.408 | 1.0585 | 0.000421 |
| PC(18:1(9Z)e/2:0) | 1.6989 | 1.5848 | 0.9328 | 0.000423 |
| Lumichrome | 1.4069 | 1.3232 | 1.0651 | 0.000424 |
