# Supplementary material for: Integrated analysis of fecal microbiome and serum metabolome reveals the profiling of gut microbiota-related metabolites in rats and mice subjected to prolonged exposure to a high-humidity environment
Source: Front Cell Infect Microbiol. 2026 Jun 22;16:1782615. doi: 10.3389/fcimb.2026.1782615 (PMC13333707; doi:10.3389/fcimb.2026.1782615)
Supplement: Supplementary file 3 [file Table2.docx]

Table S2 The top 50 differential metabolites in rat serum between W14 and Control group.

| Metabolite | VIP_pred_OPLS-DA | VIP_PLS-DA | FC(W14/con) | P_value |
| --- | --- | --- | --- | --- |
| Lumichrome | 1.655908 | 1.620445 | 1.109403 | 1.04E-07 |
| Methyl isobutyl ketone | 1.353579 | 1.327369 | 0.918946 | 1.78E-07 |
| Choline | 1.689416 | 1.649282 | 1.067984 | 3.47E-07 |
| PC(18:1(11Z)/22:6(4Z,7Z,10Z,13Z,16Z,19Z)) | 1.842177 | 1.79666 | 0.908023 | 3.51E-07 |
| PE(22:5(4Z,7Z,10Z,13Z,16Z)/22:6(4Z,7Z,10Z,13Z,16Z,19Z)) | 1.730387 | 1.694387 | 0.935132 | 4.72E-07 |
| Taurine | 1.619975 | 1.580184 | 1.083649 | 6.90E-07 |
| S-Cysteinosuccinic acid | 1.287248 | 1.26258 | 0.950298 | 7.69E-07 |
| Pantothenic Acid | 1.400617 | 1.380089 | 1.053468 | 1.78E-06 |
| 6-Methylquinoline | 1.580816 | 1.554326 | 1.069257 | 1.92E-06 |
| Isoquinoline | 1.594194 | 1.564754 | 1.08434 | 2.02E-06 |
| Indole-3-carboxaldehyde | 1.875301 | 1.826862 | 1.137533 | 2.07E-06 |
| PC(14:0/18:1(11Z)) | 2.175643 | 2.121584 | 0.869096 | 2.35E-06 |
| Betaine aldehyde | 1.563267 | 1.534398 | 1.079886 | 2.47E-06 |
| 4-formyl Indole | 1.535238 | 1.507581 | 1.06219 | 2.82E-06 |
| Benzaldehyde | 1.578944 | 1.541874 | 1.09074 | 2.95E-06 |
| PC(15:0/20:2(11Z,14Z)) | 1.758693 | 1.73583 | 0.900942 | 3.28E-06 |
| Phosphocholine | 1.526725 | 1.491366 | 1.067854 | 3.42E-06 |
| PC(16:0/18:2(9Z,12Z)) | 1.546549 | 1.515131 | 0.947293 | 3.88E-06 |
| PA(18:3(6Z,9Z,12Z)/22:6(4Z,7Z,10Z,13Z,16Z,19Z)) | 1.632527 | 1.600612 | 0.923906 | 4.28E-06 |
| DG(20:5(5Z,8Z,11Z,14Z,17Z)/22:5(4Z,7Z,10Z,13Z,16Z)/0:0) | 1.43008 | 1.395492 | 0.954984 | 4.30E-06 |
| 2-Methylbutyroylcarnitine | 1.546968 | 1.546432 | 1.069836 | 5.40E-06 |
| PS(18:2(9Z,12Z)/24:0) | 1.679588 | 1.636964 | 0.921897 | 6.18E-06 |
| Dihydrocoumarin | 1.345781 | 1.308685 | 1.044952 | 6.37E-06 |
| PC(15:0/22:4(7Z,10Z,13Z,16Z)) | 1.746485 | 1.696557 | 0.915538 | 7.93E-06 |
| Thymine | 1.546001 | 1.510913 | 1.088455 | 8.00E-06 |
| 4-Chlorobenzaldehyde | 1.400266 | 1.366942 | 1.056309 | 8.62E-06 |
| Galegine | 1.362106 | 1.33214 | 1.073358 | 8.71E-06 |
| PC(18:0/18:2(9Z,12Z)) | 1.591609 | 1.551363 | 0.938868 | 9.13E-06 |
| Indole | 1.483761 | 1.449483 | 1.054222 | 9.59E-06 |
| 4-Hydroxyretinoic acid | 1.365545 | 1.336509 | 0.949248 | 1.03E-05 |
| PE(14:1(9Z)/20:0) | 1.512657 | 1.481908 | 0.94167 | 1.04E-05 |
| Tridecanoylglycine | 1.310409 | 1.281671 | 1.06449 | 1.07E-05 |
| PC(16:0/18:3(6Z,9Z,12Z)) | 1.760358 | 1.711477 | 0.919266 | 1.14E-05 |
| Pectachol | 1.240567 | 1.230849 | 0.950556 | 1.25E-05 |
| PC(18:0/20:4(8Z,11Z,14Z,17Z)) | 1.585125 | 1.547949 | 0.946025 | 1.28E-05 |
| PS(15:0/20:5(5Z,8Z,11Z,14Z,17Z)) | 1.474801 | 1.446352 | 0.93759 | 1.34E-05 |
| Uracil | 1.557432 | 1.521044 | 1.087938 | 1.41E-05 |
| PC(16:0/18:1(11Z)) | 1.655613 | 1.618869 | 0.927994 | 1.43E-05 |
| LysoPC(16:1(9Z)) | 1.331003 | 1.297952 | 0.95734 | 1.43E-05 |
| PC(14:0/18:2(9Z,12Z)) | 1.805321 | 1.763778 | 0.904503 | 1.50E-05 |
| Diplosporin | 1.164906 | 1.144283 | 1.048768 | 1.53E-05 |
| PE-NMe2(22:5(4Z,7Z,10Z,13Z,16Z)/24:1(15Z)) | 1.72193 | 1.680604 | 0.910895 | 1.55E-05 |
| 4-HYDROXY-6-METHYLPYRAN-2-ONE | 1.395499 | 1.353506 | 1.066255 | 1.65E-05 |
| 4-Phenyl-2-butanol | 1.508561 | 1.473079 | 1.079104 | 1.83E-05 |
| (S)-(-)-Perillyl alcohol | 1.430413 | 1.399768 | 1.077379 | 1.87E-05 |
| PC(15:0/18:2(9Z,12Z)) | 1.572489 | 1.555267 | 0.921672 | 1.96E-05 |
| Solasodine | 1.572621 | 1.533031 | 0.909778 | 1.97E-05 |
| PC(16:1(9Z)/22:6(4Z,7Z,10Z,13Z,16Z,19Z)) | 1.896587 | 1.847959 | 0.886425 | 2.03E-05 |
| PC(16:0/20:4(5Z,8Z,11Z,14Z)) | 1.611878 | 1.575927 | 0.943143 | 2.06E-05 |
| INDOLE-3-CARBINOL | 1.840315 | 1.795401 | 1.134796 | 2.06E-05 |
