# Supplementary material for: Integrated analysis of fecal microbiome and serum metabolome reveals the profiling of gut microbiota-related metabolites in rats and mice subjected to prolonged exposure to a high-humidity environment
Source: Front Cell Infect Microbiol. 2026 Jun 22;16:1782615. doi: 10.3389/fcimb.2026.1782615 (PMC13333707; doi:10.3389/fcimb.2026.1782615)
Supplement: Supplementary file 4 [file Table3.docx]

Table S3 The top 50 differential metabolites in rat serum between W28 and Control group.

| Metabolite | VIP_pred_OPLS-DA | VIP_PLS-DA | FC(W28/con) | P_value |
| --- | --- | --- | --- | --- |
| Taurine | 1.515344 | 1.469484 | 1.086256 | 2.45E-07 |
| S-Cysteinosuccinic acid | 1.247521 | 1.209908 | 0.944997 | 3.28E-07 |
| Cuminaldehyde | 1.716127 | 1.673632 | 1.147249 | 8.42E-07 |
| Isoquinoline | 1.65606 | 1.598055 | 1.105365 | 8.99E-07 |
| PC(18:1(11Z)/22:6(4Z,7Z,10Z,13Z,16Z,19Z)) | 1.764394 | 1.717611 | 0.89769 | 1.23E-06 |
| LysoPC(16:0) | 1.717994 | 1.677759 | 0.89273 | 1.37E-06 |
| PC(17:1(9Z)/0:0) | 1.338185 | 1.303515 | 0.937582 | 1.38E-06 |
| PS(18:2(9Z,12Z)/24:0) | 1.699232 | 1.662583 | 0.903935 | 1.41E-06 |
| 6-Methylquinoline | 1.609728 | 1.548354 | 1.082947 | 1.64E-06 |
| PE(22:5(4Z,7Z,10Z,13Z,16Z)/22:6(4Z,7Z,10Z,13Z,16Z,19Z)) | 1.678328 | 1.624252 | 0.927746 | 1.90E-06 |
| PC(15:0/20:2(11Z,14Z)) | 1.889735 | 1.841986 | 0.867617 | 1.91E-06 |
| 4-formyl Indole | 1.581119 | 1.517676 | 1.075925 | 2.42E-06 |
| PC(18:0/18:2(9Z,12Z)) | 1.552767 | 1.511321 | 0.930811 | 2.48E-06 |
| PC(14:0/18:1(11Z)) | 2.08948 | 2.026219 | 0.855252 | 2.54E-06 |
| Betaine aldehyde | 1.611285 | 1.546374 | 1.098193 | 2.70E-06 |
| PC(15:0/18:2(9Z,12Z)) | 1.71314 | 1.659066 | 0.89549 | 3.02E-06 |
| Pectachol | 1.190215 | 1.194486 | 0.945566 | 3.13E-06 |
| Methyl isobutyl ketone | 1.242041 | 1.210934 | 0.91631 | 3.39E-06 |
| PC(16:0/18:3(6Z,9Z,12Z)) | 1.751164 | 1.70773 | 0.904141 | 3.50E-06 |
| PC(16:0/18:2(9Z,12Z)) | 1.462878 | 1.414407 | 0.944258 | 4.31E-06 |
| PC(18:0/22:6(4Z,7Z,10Z,13Z,16Z,19Z)) | 1.521679 | 1.477384 | 0.930891 | 4.57E-06 |
| PC(16:0/18:1(11Z)) | 1.588263 | 1.541492 | 0.922166 | 4.80E-06 |
| PE(14:1(9Z)/20:0) | 1.468816 | 1.421118 | 0.935837 | 4.96E-06 |
| Benzaldehyde | 1.520001 | 1.458497 | 1.098989 | 5.28E-06 |
| Choline | 1.529561 | 1.508515 | 1.068518 | 6.00E-06 |
| Galegine | 1.276186 | 1.234523 | 1.075917 | 6.48E-06 |
| PC(18:0/20:4(8Z,11Z,14Z,17Z)) | 1.469291 | 1.421359 | 0.945252 | 7.83E-06 |
| Solasodine | 1.477237 | 1.425659 | 0.90691 | 7.88E-06 |
| L-Tryptophan | 1.177107 | 1.148021 | 1.045491 | 9.00E-06 |
| Indole | 1.331955 | 1.290256 | 1.052089 | 1.09E-05 |
| Tridecanoylglycine | 1.198731 | 1.159025 | 1.06395 | 1.23E-05 |
| 4-Chlorobenzaldehyde | 1.23955 | 1.199231 | 1.052972 | 1.23E-05 |
| PC(19:1(9Z)/0:0) | 1.45749 | 1.41724 | 0.921695 | 1.29E-05 |
| 2,6,10,10-Tetramethyl-1-oxaspiro[4.5]decan-6-ol | 1.19795 | 1.153846 | 1.050994 | 1.38E-05 |
| Diplosporin | 1.107824 | 1.062941 | 1.0508 | 1.40E-05 |
| P-Salicylic acid | 1.352917 | 1.299418 | 1.095072 | 1.46E-05 |
| Phosphocholine | 1.444741 | 1.382214 | 1.071671 | 1.56E-05 |
| Thymine | 1.383429 | 1.338868 | 1.084981 | 1.61E-05 |
| PC(16:0/22:4(7Z,10Z,13Z,16Z)) | 1.52122 | 1.471839 | 0.926275 | 1.69E-05 |
| (S)-(-)-Perillyl alcohol | 1.321684 | 1.277193 | 1.078188 | 1.98E-05 |
| N-(2-Phenylethyl)-acetamide | 1.358161 | 1.30992 | 1.083585 | 1.98E-05 |
| Citreoviridin C | 1.234642 | 1.191768 | 0.94623 | 2.07E-05 |
| PE-NMe2(22:5(4Z,7Z,10Z,13Z,16Z)/24:1(15Z)) | 1.583716 | 1.53012 | 0.910248 | 2.11E-05 |
| INDOLE-3-CARBINOL | 1.675514 | 1.61769 | 1.132516 | 2.11E-05 |
| PC(16:0/20:4(5Z,8Z,11Z,14Z)) | 1.511707 | 1.460154 | 0.940644 | 2.18E-05 |
| Creatine | 1.843353 | 1.765492 | 1.099128 | 2.40E-05 |
| Sphinganine 1-phosphate | 1.124613 | 1.093356 | 0.959903 | 2.44E-05 |
| Scyphostatin A | 1.668596 | 1.612255 | 0.881559 | 2.61E-05 |
| Phenylacetaldehyde | 1.636171 | 1.56382 | 1.119314 | 2.72E-05 |
| 4-HYDROXY-6-METHYLPYRAN-2-ONE | 1.343181 | 1.279959 | 1.072682 | 2.79E-05 |
