# Supplementary material for: Integrated analysis of fecal microbiome and serum metabolome reveals the profiling of gut microbiota-related metabolites in rats and mice subjected to prolonged exposure to a high-humidity environment
Source: Front Cell Infect Microbiol. 2026 Jun 22;16:1782615. doi: 10.3389/fcimb.2026.1782615 (PMC13333707; doi:10.3389/fcimb.2026.1782615)
Supplement: Supplementary file 5 [file Table4.docx]

Table S4 The top 50 differential metabolites in mouse serum between W7 and Control group.

| Metabolite | VIP_pred_OPLS-DA | VIP_PLS-DA | FC(W7/con) | P_value |
| --- | --- | --- | --- | --- |
| Bis(2-ethylhexyl) phthalate | 1.296666 | 1.242104 | 0.97165 | 1.62E-06 |
| D-Pipecolic acid | 1.884761 | 1.808172 | 1.075173 | 6.32E-06 |
| Ne-Methyl-L-lysine | 1.688299 | 1.609621 | 1.082611 | 8.28E-06 |
| Choline | 1.727867 | 1.645795 | 1.04834 | 9.28E-06 |
| LPC(18:1) | 1.455349 | 1.36547 | 0.965384 | 1.54E-05 |
| DL-Ornithino-L-alanine | 1.271434 | 1.298761 | 1.035483 | 2.96E-05 |
| PE(17:0/0:0) | 1.841986 | 1.763663 | 0.918992 | 3.00E-05 |
| PC(20:2(11Z,14Z)/22:6(4Z,7Z,10Z,13Z,16Z,19Z)) | 2.021788 | 1.91812 | 0.921569 | 6.98E-05 |
| Alpha-Zearalenol | 1.30913 | 1.244657 | 0.955276 | 7.83E-05 |
| 2E,6E,8E-decatrienoic acid | 1.775796 | 1.70123 | 1.09704 | 0.000107 |
| PC(18:1(11Z)/22:6(4Z,7Z,10Z,13Z,16Z,19Z)) | 1.96643 | 1.851243 | 0.925626 | 0.000108 |
| PC(14:0/18:2(9Z,12Z)) | 2.016045 | 1.896641 | 0.913558 | 0.000111 |
| Sonchifolin | 1.518589 | 1.444452 | 0.954329 | 0.000115 |
| 4-Hydroxybenzaldehyde | 1.721547 | 1.647551 | 1.068862 | 0.00012 |
| 2-Hydroxycinnamic acid | 1.735173 | 1.658464 | 1.062603 | 0.000132 |
| PC(18:3(6Z,9Z,12Z)/18:3(6Z,9Z,12Z))[U] | 1.919487 | 1.809608 | 0.9322 | 0.000139 |
| Scyphostatin A | 2.365536 | 2.308996 | 0.857778 | 0.000145 |
| LPE(18:2) | 1.276162 | 1.278685 | 0.974476 | 0.000146 |
| LysoPE(0:0/18:1(11Z)) | 2.465561 | 2.378895 | 0.857766 | 0.000158 |
| Phosphocholine | 1.626861 | 1.531793 | 1.047223 | 0.000177 |
| L-Lysine | 1.645365 | 1.574991 | 1.068022 | 0.000198 |
| PC(18:1(11Z)/18:3(6Z,9Z,12Z)) | 1.836869 | 1.744555 | 0.938701 | 0.000201 |
| Xestoaminol C | 1.608577 | 1.498318 | 1.05515 | 0.000232 |
| L-Proline | 1.352397 | 1.299754 | 1.04319 | 0.000234 |
| LysoPE(20:1(11Z)/0:0) | 1.391739 | 1.417263 | 0.943454 | 0.000241 |
| 2,5-Dimethylbenzaldehyde | 1.714536 | 1.61872 | 1.093016 | 0.000253 |
| LPC(16:0) | 1.540132 | 1.474954 | 1.038895 | 0.000266 |
| Cysteinyl-Valine | 1.592581 | 1.505598 | 1.059629 | 0.000301 |
| Phenylacetaldehyde | 1.843148 | 1.769172 | 1.099297 | 0.000329 |
| Sucrose | 1.433768 | 1.372589 | 0.951864 | 0.00033 |
| Indole | 1.754631 | 1.677501 | 1.057432 | 0.000332 |
| Melleolide | 1.463226 | 1.38208 | 0.958132 | 0.000341 |
| Pantothenic Acid | 1.457498 | 1.364996 | 1.044622 | 0.000354 |
| Acrimarine J | 1.392652 | 1.411889 | 0.956767 | 0.000362 |
| PE(18:0/0:0) | 1.380975 | 1.322724 | 0.96417 | 0.00038 |
| PC(16:0/18:2(9Z,12Z)) | 1.760017 | 1.63707 | 0.952187 | 0.000384 |
| 1-Phenyl-1-propanol | 1.776673 | 1.676987 | 1.084896 | 0.000419 |
| Kanzonol M | 1.422842 | 1.335875 | 0.963614 | 0.000449 |
| 6-Methylquinoline | 1.999325 | 1.899908 | 1.085216 | 0.00047 |
| PC(16:0/18:3(9Z,12Z,15Z)) | 1.84266 | 1.708103 | 0.939038 | 0.000474 |
| 5-Hydroxyindoleacetic acid | 1.534825 | 1.480409 | 1.064171 | 0.000486 |
| Dihydrocoumarin | 1.49083 | 1.434268 | 1.043598 | 0.000492 |
| 4-Chlorobenzaldehyde | 1.522857 | 1.459191 | 1.051981 | 0.0005 |
| Benzaldehyde | 1.615718 | 1.557883 | 1.073897 | 0.000515 |
| PC(18:1(11Z)/20:4(5Z,8Z,11Z,14Z)) | 1.792438 | 1.650168 | 0.941264 | 0.000535 |
| 4-Pyrimidine Methanamine (hydrochloride) | 1.605591 | 1.532527 | 1.076175 | 0.000674 |
| 4-formyl Indole | 1.959419 | 1.862599 | 1.07868 | 0.000679 |
| (S)-(-)-Perillyl alcohol | 1.573953 | 1.504912 | 1.07034 | 0.000701 |
| 4-HYDROXY-6-METHYLPYRAN-2-ONE | 1.607762 | 1.49647 | 1.063908 | 0.000704 |
| Melibiose | 1.168091 | 1.098966 | 0.975211 | 0.000787 |
