# Supplementary material for: Integrated analysis of fecal microbiome and serum metabolome reveals the profiling of gut microbiota-related metabolites in rats and mice subjected to prolonged exposure to a high-humidity environment
Source: Front Cell Infect Microbiol. 2026 Jun 22;16:1782615. doi: 10.3389/fcimb.2026.1782615 (PMC13333707; doi:10.3389/fcimb.2026.1782615)
Supplement: Supplementary file 6 [file Table5.docx]

Table S4 The top 50 differential metabolites in mouse serum between W14 and Control group.

| Metabolite | VIP_pred_OPLS-DA | VIP_PLS-DA | FC(W14/con) | P_value |
| --- | --- | --- | --- | --- |
| Bis(2-ethylhexyl) phthalate | 1.14195 | 1.119774 | 0.971109 | 4.00E-08 |
| LPC(18:1) | 1.344029 | 1.318345 | 0.96064 | 3.36E-07 |
| Alpha-Zearalenol | 1.205976 | 1.198139 | 0.950187 | 1.22E-06 |
| Choline | 1.601524 | 1.56963 | 1.055393 | 1.35E-06 |
| Glycerophosphocholine | 1.584331 | 1.553279 | 1.09434 | 1.58E-06 |
| D-Pipecolic acid | 1.707729 | 1.690801 | 1.083426 | 1.58E-06 |
| 2E,6E,8E-decatrienoic acid | 1.74206 | 1.716403 | 1.118096 | 2.15E-06 |
| PE(17:0/0:0) | 1.531431 | 1.500447 | 0.927636 | 2.50E-06 |
| PC(14:0/18:2(9Z,12Z)) | 1.919227 | 1.860852 | 0.898816 | 2.91E-06 |
| N-Methyl-1-deoxynojirimycin | 1.713228 | 1.676078 | 1.118933 | 3.27E-06 |
| PC(20:2(11Z,14Z)/22:6(4Z,7Z,10Z,13Z,16Z,19Z)) | 1.854846 | 1.818167 | 0.91451 | 3.33E-06 |
| Melibiose | 1.060624 | 1.03325 | 0.975035 | 5.25E-06 |
| PC(18:1(11Z)/18:3(6Z,9Z,12Z)) | 1.755328 | 1.703777 | 0.930178 | 5.83E-06 |
| LPC(16:0) | 1.580031 | 1.535785 | 1.049849 | 6.31E-06 |
| Kanzonol M | 1.282138 | 1.245089 | 0.963259 | 8.46E-06 |
| PC(18:1(11Z)/22:6(4Z,7Z,10Z,13Z,16Z,19Z)) | 1.812967 | 1.770623 | 0.917341 | 8.98E-06 |
| Sonchifolin | 1.36769 | 1.335976 | 0.952542 | 1.06E-05 |
| PC(16:1(9Z)/22:6(4Z,7Z,10Z,13Z,16Z,19Z)) | 1.731056 | 1.673974 | 0.923515 | 1.18E-05 |
| PC(18:3(6Z,9Z,12Z)/18:3(6Z,9Z,12Z))[U] | 1.784363 | 1.728679 | 0.923121 | 1.18E-05 |
| 4-HYDROXY-6-METHYLPYRAN-2-ONE | 1.500146 | 1.472838 | 1.071254 | 1.25E-05 |
| Phosphocholine | 1.605369 | 1.558387 | 1.058983 | 1.32E-05 |
| Cysteinyl-Valine | 1.606476 | 1.564732 | 1.076798 | 1.78E-05 |
| PC(16:0/18:3(9Z,12Z,15Z)) | 1.721316 | 1.656474 | 0.930835 | 2.98E-05 |
| 2-Hydroxycinnamic acid | 1.52821 | 1.505165 | 1.063427 | 3.13E-05 |
| Spermidine | 1.748341 | 1.707732 | 1.101301 | 3.27E-05 |
| 4-Hydroxybenzaldehyde | 1.574284 | 1.552309 | 1.075541 | 3.32E-05 |
| PC(18:1(11Z)/20:4(5Z,8Z,11Z,14Z)) | 1.707311 | 1.65059 | 0.930415 | 3.71E-05 |
| Melleolide | 1.28769 | 1.245621 | 0.958904 | 4.02E-05 |
| 1-Phenyl-1-propanol | 1.648419 | 1.616142 | 1.09534 | 4.29E-05 |
| Taurine | 1.439475 | 1.45235 | 1.063811 | 4.33E-05 |
| (S)-(-)-Perillyl alcohol | 1.615415 | 1.581081 | 1.091468 | 4.34E-05 |
| C16 Sphinganine | 1.457868 | 1.429071 | 1.050626 | 4.36E-05 |
| PC(18:2(9Z,12Z)/22:6(4Z,7Z,10Z,13Z,16Z,19Z)) | 1.653831 | 1.632431 | 0.933806 | 4.59E-05 |
| LysoPE(0:0/18:1(11Z)) | 2.432259 | 2.373497 | 0.815847 | 4.62E-05 |
| 4-Pyrimidine Methanamine (hydrochloride) | 1.554657 | 1.547759 | 1.091743 | 4.65E-05 |
| Ectocarpen | 1.709701 | 1.676177 | 1.106307 | 4.88E-05 |
| Xestoaminol C | 1.370277 | 1.344151 | 1.05515 | 5.06E-05 |
| Phenylacetaldehyde | 1.737047 | 1.70553 | 1.112236 | 5.74E-05 |
| Indole | 1.708229 | 1.663414 | 1.068449 | 5.77E-05 |
| PC(18:0/18:3(9Z,12Z,15Z)) | 1.596014 | 1.569535 | 0.939 | 6.47E-05 |
| Styrene | 1.52242 | 1.486303 | 1.055204 | 6.94E-05 |
| Sucrose | 1.277677 | 1.231966 | 0.952316 | 7.31E-05 |
| L-Lysine | 1.459098 | 1.461393 | 1.074046 | 7.32E-05 |
| PC(17:2(9Z,12Z)/0:0) | 1.564915 | 1.510705 | 0.915557 | 7.97E-05 |
| L-Proline | 1.189004 | 1.182779 | 1.044333 | 8.19E-05 |
| (+/-)-3-[(2-methyl-3-furyl)thio]-2-butanone | 1.460514 | 1.448011 | 1.12673 | 8.58E-05 |
| Benzylazanium | 1.437431 | 1.403419 | 1.051803 | 8.65E-05 |
| Phenylacetylglycine | 1.567917 | 1.549747 | 1.0984 | 8.97E-05 |
| 2,5-Dimethylbenzaldehyde | 1.591076 | 1.567359 | 1.108254 | 9.06E-05 |
| 4-Formylsalicylic acid | 1.212582 | 1.181565 | 1.044151 | 9.20E-05 |
