# Supplementary material for: Integrated analysis of fecal microbiome and serum metabolome reveals the profiling of gut microbiota-related metabolites in rats and mice subjected to prolonged exposure to a high-humidity environment
Source: Front Cell Infect Microbiol. 2026 Jun 22;16:1782615. doi: 10.3389/fcimb.2026.1782615 (PMC13333707; doi:10.3389/fcimb.2026.1782615)
Supplement: Supplementary file 7 [file Table6.docx]

Table S6 The top 50 differential metabolites in mouse serum between W28 and Control group.

| Metabolite | VIP_pred_OPLS-DA | VIP_PLS-DA | FC(W28/con) | P_value |
| --- | --- | --- | --- | --- |
| Bis(2-ethylhexyl) phthalate | 1.115949 | 1.099011 | 0.97002528 | 7.03E-10 |
| LPC(18:1) | 1.259135 | 1.233175 | 0.96222105 | 4.67E-07 |
| D-Pipecolic acid | 1.763615 | 1.743394 | 1.09569485 | 4.69E-07 |
| N-Methyl-1-deoxynojirimycin | 1.762024 | 1.71196 | 1.1340405 | 1.13E-06 |
| PC(14:0/18:2(9Z,12Z)) | 1.885647 | 1.831704 | 0.8936788 | 2.28E-06 |
| Choline | 1.652228 | 1.598873 | 1.0627903 | 2.30E-06 |
| PC(18:3(6Z,9Z,12Z)/18:3(6Z,9Z,12Z))[U] | 1.86505 | 1.815103 | 0.90960015 | 2.46E-06 |
| PC(18:1(11Z)/18:3(6Z,9Z,12Z)) | 1.625831 | 1.57384 | 0.93543707 | 3.26E-06 |
| PE(17:0/0:0) | 1.435139 | 1.434865 | 0.92788343 | 3.62E-06 |
| Glycerophosphocholine | 1.453837 | 1.421114 | 1.08685235 | 4.46E-06 |
| LPC(16:0) | 1.505769 | 1.450853 | 1.04826163 | 4.58E-06 |
| Ne-Methyl-L-lysine | 1.446652 | 1.394542 | 1.08694307 | 4.71E-06 |
| PC(20:2(11Z,14Z)/22:6(4Z,7Z,10Z,13Z,16Z,19Z)) | 1.787905 | 1.738711 | 0.91411765 | 6.06E-06 |
| 2E,6E,8E-decatrienoic acid | 1.713887 | 1.692446 | 1.12664022 | 6.12E-06 |
| Cysteinyl-Valine | 1.659597 | 1.595468 | 1.08584687 | 8.00E-06 |
| LysoPE(20:3(5Z,8Z,11Z)/0:0) | 1.409314 | 1.383085 | 0.93334932 | 8.46E-06 |
| L-Lysine | 1.591602 | 1.600163 | 1.09337349 | 9.86E-06 |
| Phosphocholine | 1.608092 | 1.537462 | 1.06260177 | 1.35E-05 |
| 4-Hydroxybenzaldehyde | 1.615841 | 1.579824 | 1.08452326 | 1.66E-05 |
| Alpha-Zearalenol | 1.038555 | 1.020357 | 0.95902517 | 1.69E-05 |
| Taurine | 1.399877 | 1.402731 | 1.0640318 | 2.07E-05 |
| L-Proline | 1.29895 | 1.281393 | 1.05530165 | 2.07E-05 |
| 2-Hydroxycinnamic acid | 1.548975 | 1.516668 | 1.07001647 | 2.21E-05 |
| PC(16:0/18:3(9Z,12Z,15Z)) | 1.62882 | 1.575333 | 0.93232662 | 2.56E-05 |
| PC(18:1(11Z)/22:6(4Z,7Z,10Z,13Z,16Z,19Z)) | 1.657597 | 1.611087 | 0.92424541 | 2.74E-05 |
| 4-HYDROXY-6-METHYLPYRAN-2-ONE | 1.42934 | 1.402349 | 1.07149853 | 2.89E-05 |
| Sonchifolin | 1.331368 | 1.275701 | 0.95194599 | 2.95E-05 |
| 1-Phenyl-1-propanol | 1.568508 | 1.518056 | 1.0918586 | 3.38E-05 |
| Kanzonol M | 1.223698 | 1.177968 | 0.96343628 | 3.38E-05 |
| Phenylacetaldehyde | 1.820277 | 1.771713 | 1.13108298 | 3.40E-05 |
| Melleolide | 1.297868 | 1.248391 | 0.95504534 | 3.66E-05 |
| Melibiose | 1.05027 | 1.011973 | 0.97292546 | 5.72E-05 |
| 4-Pyrimidine Methanamine (hydrochloride) | 1.652642 | 1.656505 | 1.11509591 | 5.86E-05 |
| 5-Aminopentanoic acid | 1.949316 | 1.88414 | 1.14157184 | 6.50E-05 |
| 4-Chlorobenzaldehyde | 1.420078 | 1.381887 | 1.06114188 | 6.78E-05 |
| LysoPE(0:0/18:1(11Z)) | 2.24676 | 2.217211 | 0.82311606 | 6.92E-05 |
| Mucronine D | 1.041322 | 1.034143 | 0.96879815 | 7.38E-05 |
| Pantothenic Acid | 1.274556 | 1.254852 | 1.0503052 | 7.42E-05 |
| 2,5-Dimethylbenzaldehyde | 1.465485 | 1.419422 | 1.0968254 | 7.89E-05 |
| Styrene | 1.45998 | 1.400509 | 1.05375566 | 8.08E-05 |
| 2-Aminoethylphosphocholate | 1.648925 | 1.616321 | 0.88564828 | 8.10E-05 |
| Dihydrocoumarin | 1.405759 | 1.389997 | 1.05389993 | 8.29E-05 |
| L-Valine | 1.685057 | 1.662146 | 1.07437722 | 8.49E-05 |
| PC(18:0/18:3(9Z,12Z,15Z)) | 1.50677 | 1.457524 | 0.94206822 | 8.73E-05 |
| (S)-(-)-Perillyl alcohol | 1.463803 | 1.408666 | 1.08050281 | 8.93E-05 |
| (+/-)-3-[(2-methyl-3-furyl)thio]-2-butanone | 1.572799 | 1.561569 | 1.16089965 | 8.95E-05 |
| Indole-3-acetamide | 1.805575 | 1.758518 | 1.14168937 | 9.49E-05 |
| Sucrose | 1.21103 | 1.164886 | 0.95322034 | 0.000104 |
| 5-Oxo-2(5H)-isoxazolepropanenitrile | 1.800017 | 1.794779 | 1.12058315 | 0.000106 |
| Benzaldehyde | 1.459072 | 1.426469 | 1.08213716 | 0.000109 |
